# Dual-version and dual-scale CSRR loaded sensor for mining-based material characterization

**DOI:** 10.1371/journal.pone.0360251

**Published:** 2026-10-09

**Authors:** Madan Kumar Sharma, Abdullah Said Alkalbani, Satyanarayana Degala, Gopal Rathinam, Madhur Deo Upadhayay

**Affiliations:** 1 College of Engineering, University of Buraimi, Oman; 2 Department of EE, Shiv Nadar University, Delhi NCR, India; SVM Arts, Science and Commerce College, INDIA

## Abstract

In this work, dual-version and dual-scale sensor is proposed for mining-based material characterization. The sensor structure is configured using a circular-shaped patch loaded with a 3 × 3 CSRR array. The ground plane was fully loaded with a perfect electric conductor. A strong resonance for the (S21) parameter is achieved by four rectangular slots created surrounding the CSRR array. The first version of the sensor had an overall size of 100 mm × 60 mm and resonated at a frequency of 1.5 GHz. Furthermore, the sensor size was optimized, and the second version achieved an overall size of 30 mm × 20 mm. The second version provides a resonating peak at 4.88 GHz for the transmission coefficient. The effectiveness of the sensing area was analyzed using the electric field distribution and current density. Both sensors are fabricated and experimentally validated by comparison of simulated and measured results. In terms of sensing applications, mining-based materials (Copper, Chrome and Silica) were tested using both sensors. The dual-scale resonating capabilities of the sensors can effectively characterize the Material Under Test (MUT). In comparison, the proposed sensors have a compact size, dual scale of measurements, and capability of characterizing mining-based materials.

## 1. Introduction

Accurate characterization of mining-based materials is critical in determining ore quality and ensuring efficient mining processes [1]. However, conventional characterization of mining-based materials is often labor-intensive, expensive, and not suitable for real-time applications [2]. To mitigate these challenges, resonator-based microwave sensor technology has been considered as a potential solution owing to its high sensitivity and non-destructive evaluation capabilities [3]. The microwave sensors provide material characterization by analyzing the interaction of electromagnetic waves with a Material Under Test (MUT) through absorption, reflection, and transmission, which are influenced by permittivity, conductivity, and permeability [4,5]. Their resonating behavior enhances the sensing accuracy, making them valuable across fields such as agriculture, biomedical, food, construction, and mining [6–8]. Among various designs, Split Ring Resonators (SRR) and Complementary SRR (CSRR) are widely used for their compact size, high sensitivity, and efficient electromagnetic control.

CSRR- and DGS-based sensors have been applied for liquid and solid characterizations, demonstrating resonance shifts with edible oil, contaminated water, and dielectric solids [9,10]. Designs such as maze-shaped and curved-shaped CSRRs enhanced sensitivity and enabled dual testing, with resonances ranging from 2.45 to 4.05 GHz [11,12]. These studies highlight the versatility of CSRR sensors for diverse material-sensing applications. Recent studies have demonstrated various resonator-based humidity and material sensors with high sensitivity. An MXene-coated meander line sensor achieved single resonance at 4.1 GHz [13], whereas Zahra and Michal introduced an SRR-loaded transmission line for dual sensing and filtering [14]. A square SRR-based sensor was integrated into a three-node wireless network to detect ice conditions [15]. Interdigit capacitive sensors operating at 2.4 GHz were used for liquid and solid testing [16], and a two-port THZ-based sensor characterized solid materials effectively [17]. Enhanced performance has also been achieved using CSRR structures, including dumbbell-shaped [18] and interdigital electrode-integrated [19] designs. Overall, the SRR and CSRR architectures enable strong field confinement in the sensing area, significantly improving the sensor sensitivity to the materials under test (MUT).

In order to provide dependable material characterization and monitoring, recent developments in sensor technology have shown the significance of multi-sensor dependency modeling, flexible sensing platforms, wireless sensor designs, and enhanced calibration techniques [20–22]. Multi-scale sensing approaches are becoming increasingly important, as evidenced by the development of high-precision cross-scale characterization techniques for complicated three-dimensional objects [23]. Additionally, sensor array accuracy and reliability have been enhanced by directionally regularized calibration techniques and physics-inspired error modeling [24]. The adaptability of modern sensing systems for high-frequency and condition-monitoring applications is further demonstrated by recent advancements in time-frequency integration architectures and planar magnetically linked current sensors [25,26]. In mining-related settings, image-processing-based techniques have also been effectively used for material identification and classification [27]. Furthermore, guided-surface lattice resonances and high-Q resonant structures have garnered significant interest because of their acute resonance features and increased sensitivity [28]. For cutting-edge industrial applications, sustainable material-based sensing and prediction techniques are also developed in [29]. The creation of the suggested dual-version and dual-scale CSRR-loaded sensor for mining-based material characterization is supported by the collective findings of this research, which demonstrate the increasing demand for small, precise, high-Q, and multi-scale sensing platforms.

Despite a comprehensive search of existing literature, no prior work has been reported in the specific domain of mining‑based material characterization employing CSRR sensors. Indeed, all the existing works on CSRR sensors have been carried out on industrial, chemical, and biological materials. Therefore, there is a limitation to execute quantitative comparison with the existing literature. However, we compared and discussed related literature of the resonating sensor with proposed work in Table 1. Instead, the novelty of the work is emphasized, and the performance evaluation is carried out in detail using the experimental results obtained.

**Table 1:** A brief comparative study with exiting methods.

Ref.	Size of the Sensor	Resonance (GHz)	Q-factor	Sensor Type	MUT	Industrial Applications
[9]	0.214 λ0 × 0.16 λ0	3.21	51.63	CSRR	Edible oil	Food
[10]	0.33 λ0 × 0.33 λ0	4.0	*	CSRR	Water Contamination	Food
[11]	0.27 λ0 × 0.27 λ0	2.88 & 4.05	780.5	CSRR	Solid Material (Roger made)	Manufacturing
[12]	0.327 λ0 × 0.327 λ0	2.45	120	CSRR	Solid & Liquid	Manufacturing
[13]	0.23 λ0 × 0.21 λ0	4.1	*	Interdigitated	Humidity	Manufacturing
[15]	0.15 λ0 × 0.09 λ0	2.5 & 3	*	SRR	Ice	Environment
[18]	0.44 λ0 × 0.19 λ0	1.89	*	CSRR	Urea in Saline	Environment
[19]	0.29 λ0 × 0.29 λ0	3.8	*	CSRR	Liquid (Ethanol &Methanol)	Environment
**This Work**	0.50 λ0 × 0.30 λ0 λ0 × 0.51 λ0	1.5 and 4.88	1219.94	Composite CSRR-Array	Solid (Minerals)	**Mining**

* Not available.

Most reported sensors in [9,10,12,13,15–17,19]operate with a single resonance, whereas only a few achieve dual [11] or triple-band performance [18]. Similarly, several designs [9,10,12,16,18,19]have been validated with liquid samples, whereas others [11,12,16,17] have focused on solids. Only a few studies [30,31] have addressed the characterization of mining-based substances. This highlights a clear research gap: the need for a versatile sensor capable of dual-scale, dual-version operation, with specific applicability to mining material characterization. In this work, a dual‑version and dual‑scale CSRR‑loaded sensor is presented. The sensor is specifically engineered to enhance the detection and differentiation of material properties commonly encountered in mining environments. The proposed sensor architecture leverages with CSRR array and slot stubs to achieve improved electromagnetic interaction with heterogeneous mining samples, thereby enabling precise and reliable material characterization.

The major contributions of this study are outlined as follows:

1. To evolve a new composite-shaped CSRR-loaded sensor.
2. An optimized version of the sensor and different operating frequency scales are explored.
3. Characterize mining-based materials (Cu, Chrome, Quartz) using dual-version and dual-scale sensors.

However, the present study is an extension of the authors’ previously published research [32]. Compared with the earlier work, this research significantly broadens its scope by introducing several novel contributions, as summarized in Table 2.

**Table 2:** Novel contribution of presented study with previously reported research by authors.

Aspect	Previous Conference/Book Chapter Publication [32]	Present Extended Work
**Research Scope**	Focused primarily on the design and simulation of the proposed sensor.	Extends the previous work with fabrication, experimental validation, and comprehensive performance assessment.
**Sensor Development**	Sensor design and performance evaluated through simulation only.	Sensor successfully fabricated and experimentally characterized.
**Validation Methodology**	Based solely on simulation results.	Includes extensive experimental investigations and validation under practical measurement conditions.
**Simulation vs. Measurement Comparison**	Not available.	Detailed comparison between simulated and measured results is presented and analysed.
**Material Characterization Study**	Material samples investigated through simulation-based analysis only.	Real mining-related material samples characterized experimentally using the fabricated sensor.
**Performance Evaluation**	Limited to simulation-based performance metrics.	Includes additional sensitivity analysis, validation studies, and performance metrics to assess robustness and practical effectiveness.
**Experimental Results**	Not included.	Comprehensive measurement results and experimental observations are reported.
**Analysis and Discussion**	Limited discussion focused on simulation outcomes.	Expanded discussion covering sensing mechanisms, experimental observations, limitations, and practical considerations.
**Practical Applicability**	Concept demonstrated theoretically through simulations.	Practical feasibility and real-world applicability demonstrated through fabrication and experimental validation.
**Novel Contributions**	Initial proof-of-concept and simulation study.	Fabrication, measurement-based validation, material characterization, extended performance analysis, and comprehensive interpretation of results.

## 2. Proposed sensor design principle

The proposed sensor is fabricated on an FR4 substrate with a dielectric constant of 4.3, a loss tangent of 0.025, and a thickness of 1.5 mm. The design layout, and dimensional specifications, is depicted in Fig 1. The sensor employs a two-port configuration with a full ground plane, and the sensing area is realized using a circular disc loaded with a composite-shaped complementary split-ring resonator (CSRR) array. A square slot surrounding the circular CSRR array enhances the resonance characteristics, yielding a strong response at approximately 1.5 GHz frequency. To facilitate measurements with powdered samples, a circular sample holder made of polyethylene terephthalate (PET) was designed, with a diameter of 56 mm and a height of 10 mm. The PET material had a dielectric constant of 2 and a loss tangent of 0.002. The simulated and measured transmission coefficient (S21) responses, with and without the sample holder, are presented in Fig 2. The results demonstrate that the inclusion of the sample holder introduces negligible variations in the S21 response, indicating minimal interference with the sensor performance. The measured results are validated by matching of the simulation results,the sensor is resonated at 1.5 GHz with S21 value −45.64 dB.

**Fig 1:**
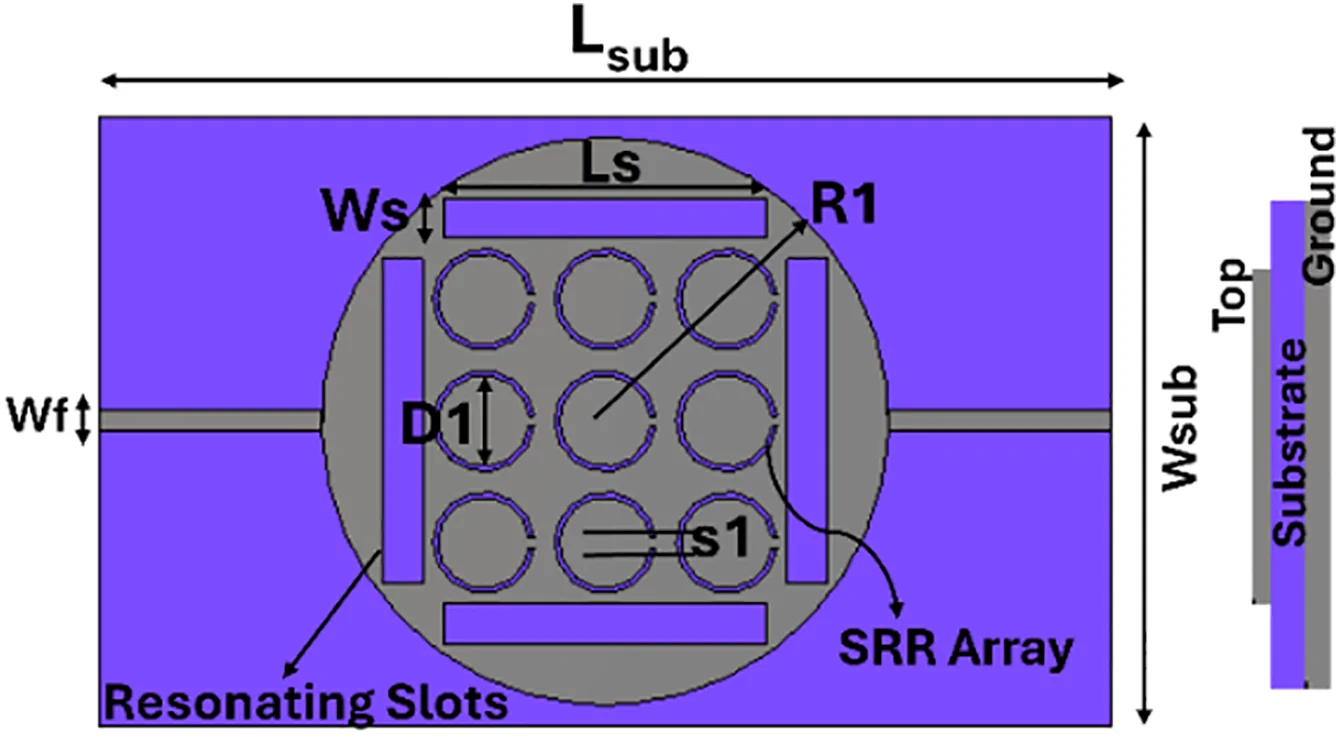
The MR-sensor (a) Without sample holder, dimensions (in mm), [Lsub = 100, Wsub = 60,Ls = 32,Ws = 4,Wf = 2,D1 = 5, s1 = 1,R1 = 14].

**Fig 2:**
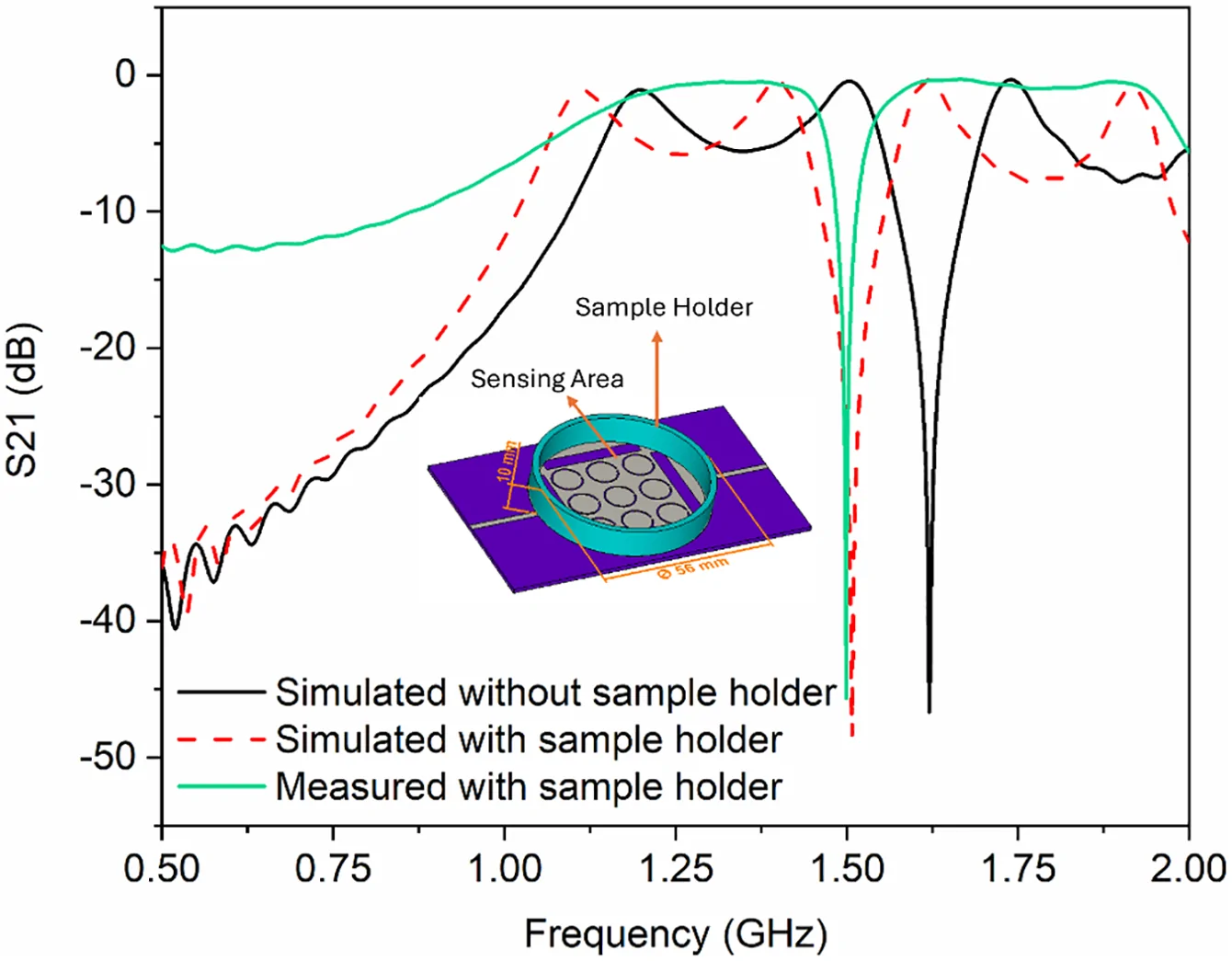
Simulated and measured transmission (S21) coefficient results of the proposed sensor with and without sample holder.

The sensor design process and the corresponding S-parameter (S21) results are presented in Fig 3. The resonance frequency is observed upon the incorporation of a CSRR array combined with surrounding rectangular slots in the sensing region, as evident from the simulation results.

**Fig 3:**
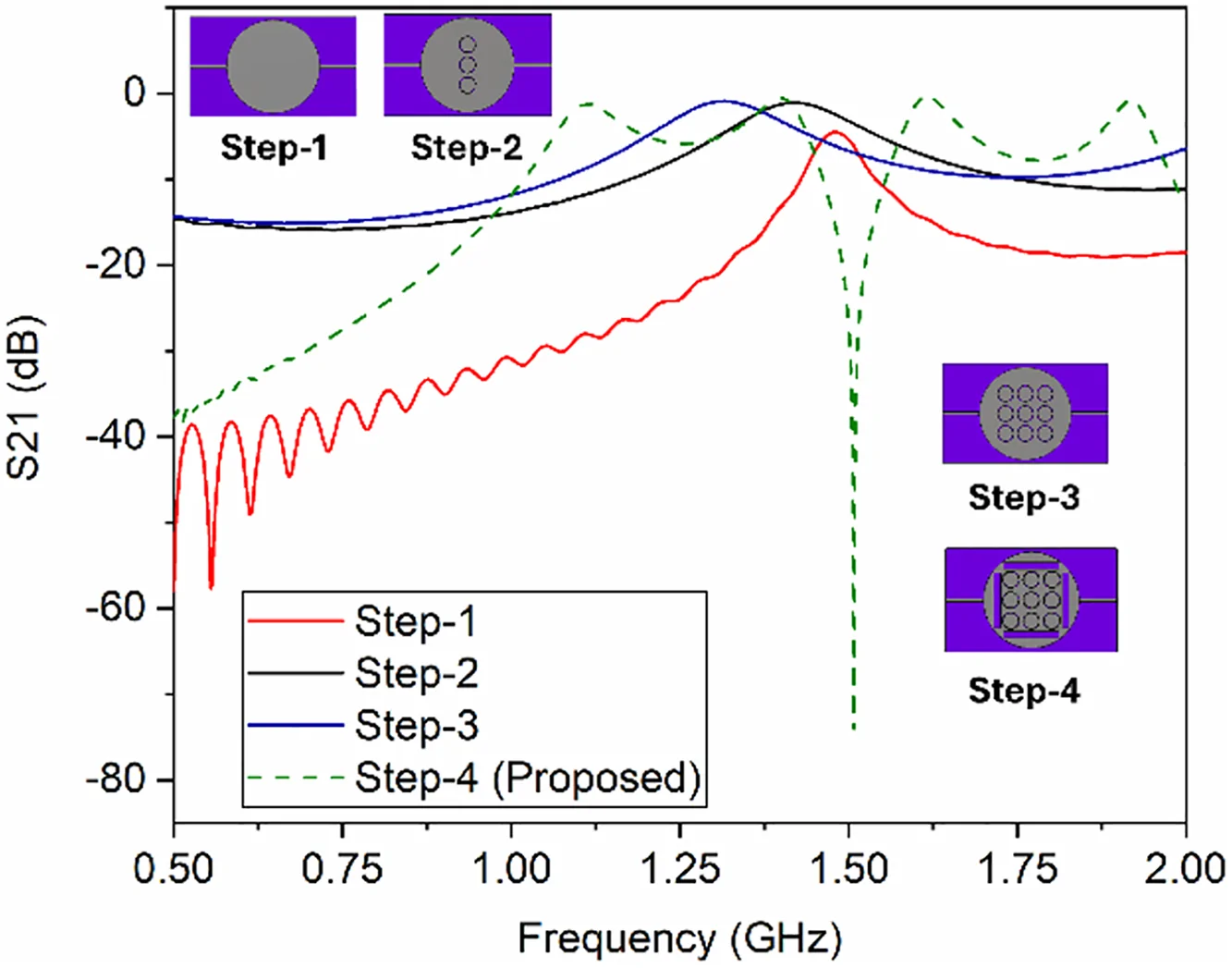
Sensor design steps and resonating behavior.

### 2.1. Resonating principle of the sensor

The resonance behavior of the sensor is governed by the distributed inductive and capacitive elements within its structure. The placement of the material under test (MUT) perturbs these properties, causing a resonance shift owing to changes in permittivity and permeability. This relationship is defined by Eqn. (1) and are detailed in [18].


$$\begin{array}{c} \frac{\Delta f_{r}}{f_{r}}=\frac{\int_{v}\left(\Delta \varepsilon E_{\text{1}}.E_{\text{0}}+\Delta \mu H_{\text{1}}.H_{\text{0}}\right)dv}{\int_{v}\left(\varepsilon_{\text{0}}{\left|E_{\text{0}}\right|}^{\text{2}}+\mu_{\text{0}}{\left|H_{\text{0}}\right|}^{\text{2}}\right)dv} \end{array}$$ (1)

Here, ∆ε and ∆μ represent the changes in permittivity and permeability, respectively, and ∆fr denotes the corresponding shift in the resonant frequency relative to the original resonant frequency fr. E_0_ and H_0_ denote the electric and magnetic field distributions under unloaded conditions, respectively, whereas E_1_ and H_1_ represent the fields when the sensor is loaded with the material under test (MUT). The field distribution within the sensing region is significantly influenced by the dielectric properties of the MUT, resulting in a measurable shift in its resonant frequency.

The electric field and current distribution of the sensor at the resonant frequency 1.5 GHz is illustrated in Fig 4. The results confirmed the resonant behavior of the structure, with the maximum field concentration observed around the CSRR array and the surrounding slotted region. The equivalent circuit model (ECM) of the sensor, based on distributed lumped elements, is presented in Fig 5. The CSRR array is modeled as a parallel LC network, where the metallic gaps between the rings function as inductors (L1 to L-6). Additionally, the four outer rectangular slots contribute to the series LC configuration. Furthermore, ECM results are validated by comparison of Computer Simulation Technology (CST) results as shown in Fig 6.

**Fig 4:**
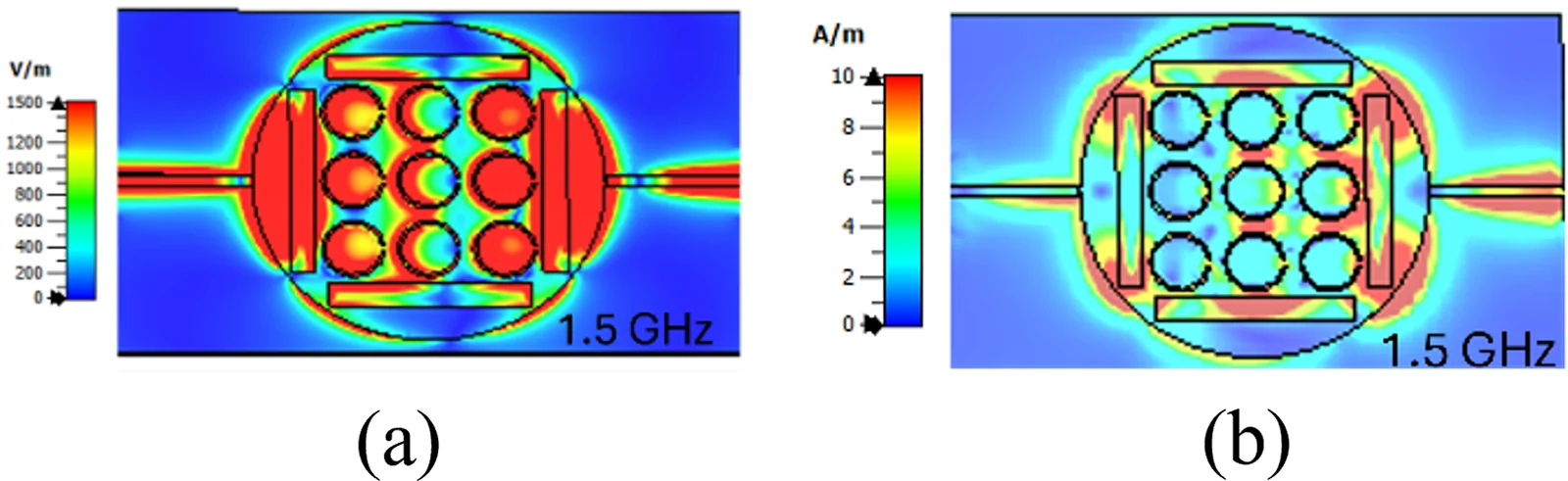
(a) Electric field distribution, and (b) Current density at 1.5 GHz.

**Fig 5:**
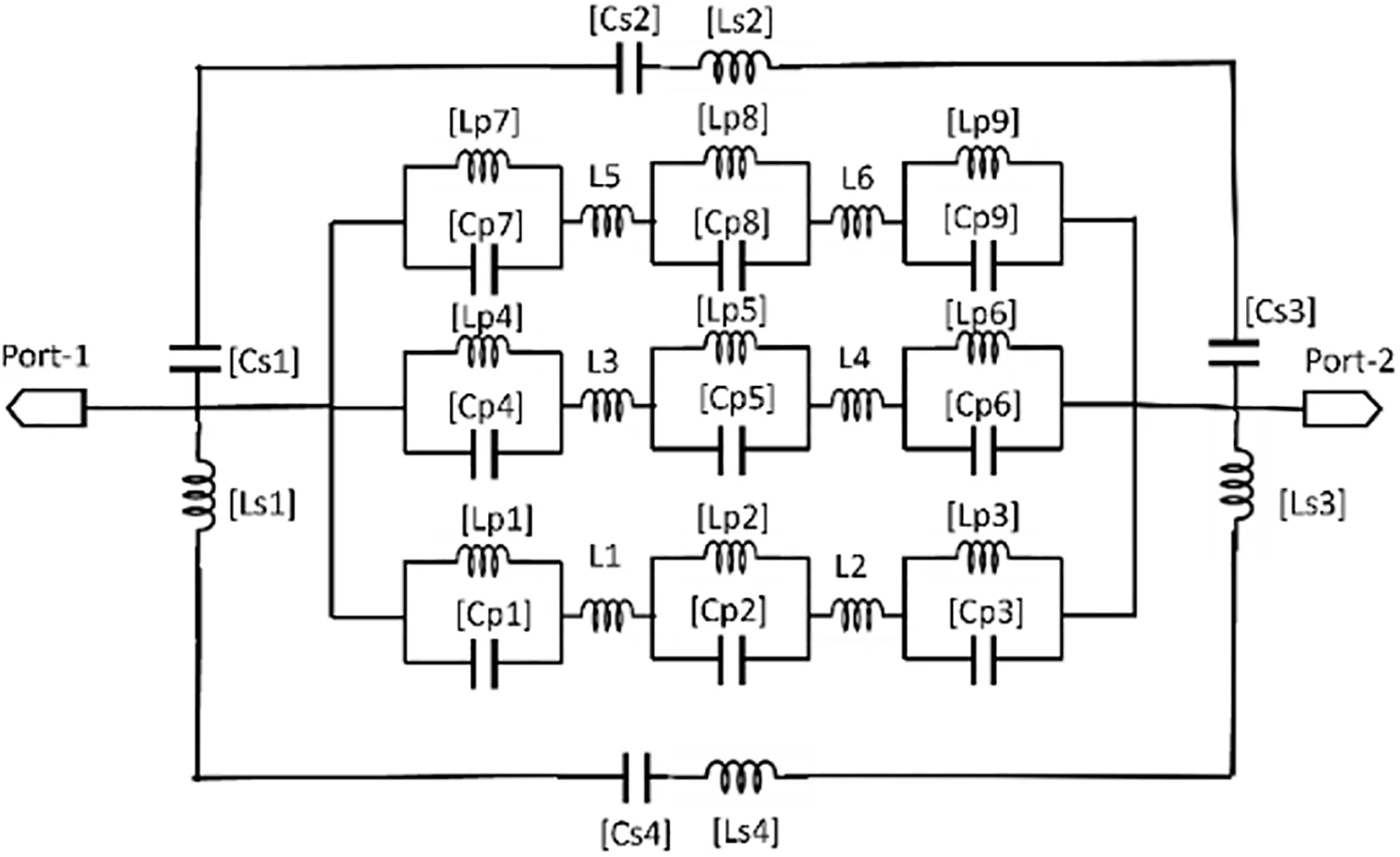
Equivalent circuit model of the proposed sensor.

**Fig 6:**
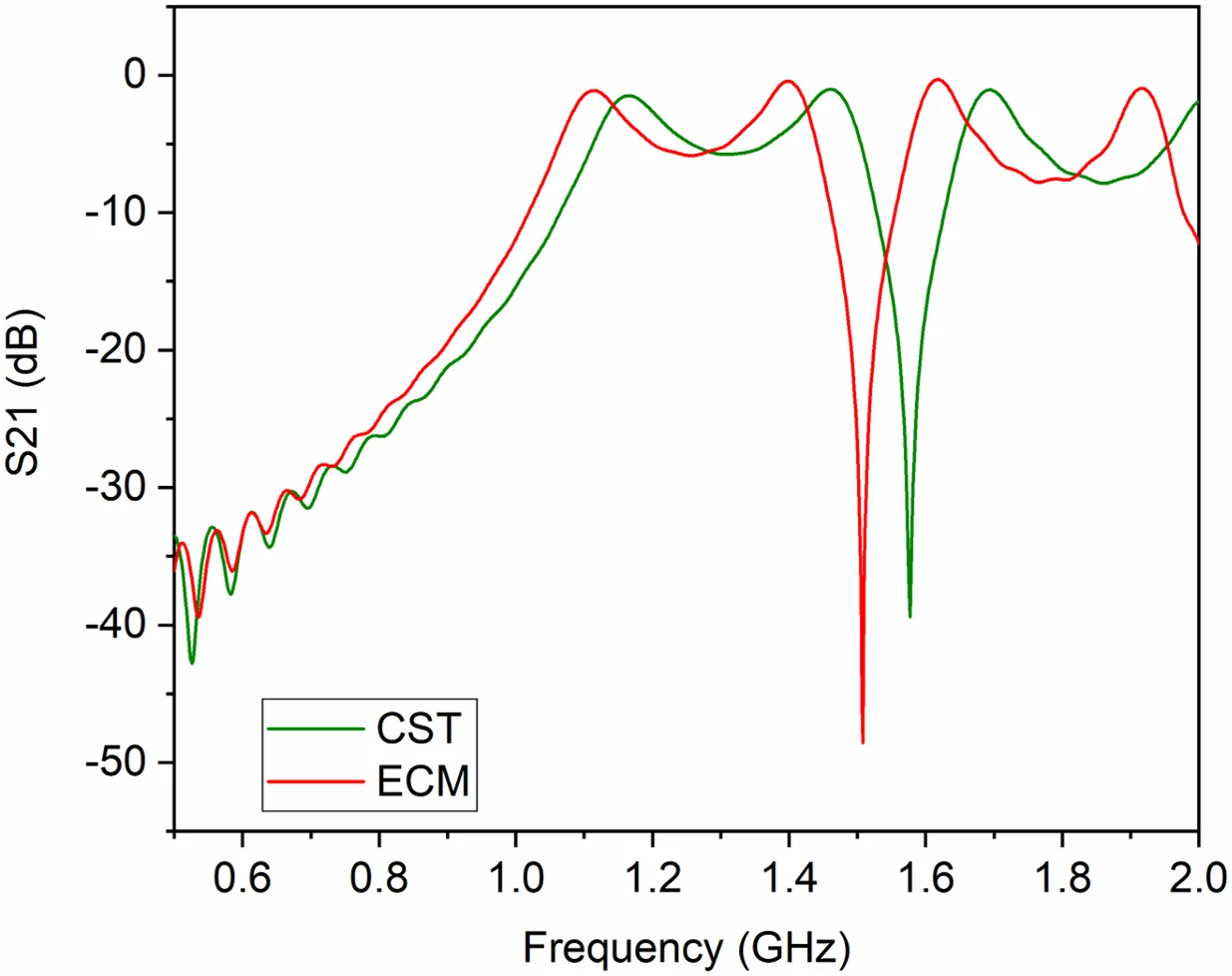
Comparison of CST and ECM Transmission results.

## 3. Mining based materials characterization

### 3.1. Sample preparation

Three mining-based materials that are prospective minerals across the geographical region of Oman were selected for this analysis. The materials used were copper, chrome, and quartz. These samples were collected from different mining companies that worked with specific minerals. The final samples were prepared by mixing different quantities (4g,8g,12g) of the purest form of mineral into the collected mining samples. Three distinct samples were prepared for each mineral category, as shown in Fig 7.

**Fig 7:**
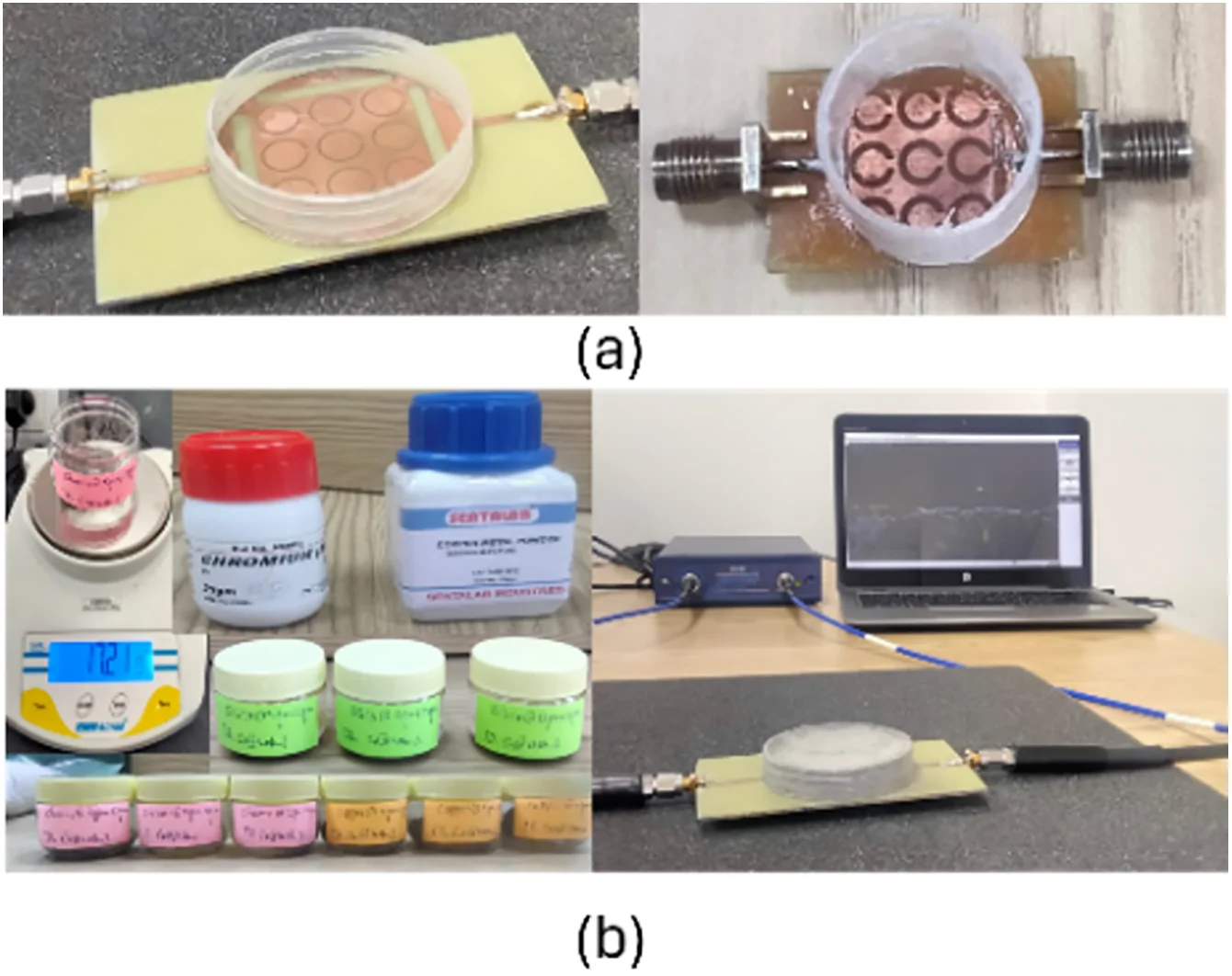
Proposed sensors and measurement (a) Proposed sensor [left side first version, right side second version] (b) Prepared samples and measurement setup.

### 3.2. Experimental analysis

The experimental analysis of the samples is carried out using first version of the proposed sensor. The transmission coefficient results for chrome, copper, and silica are shown in Fig 8. Furthermore, sensitivity of the sensor is calculated using relation [9] defined by Eq. (2).

**Fig 8:**
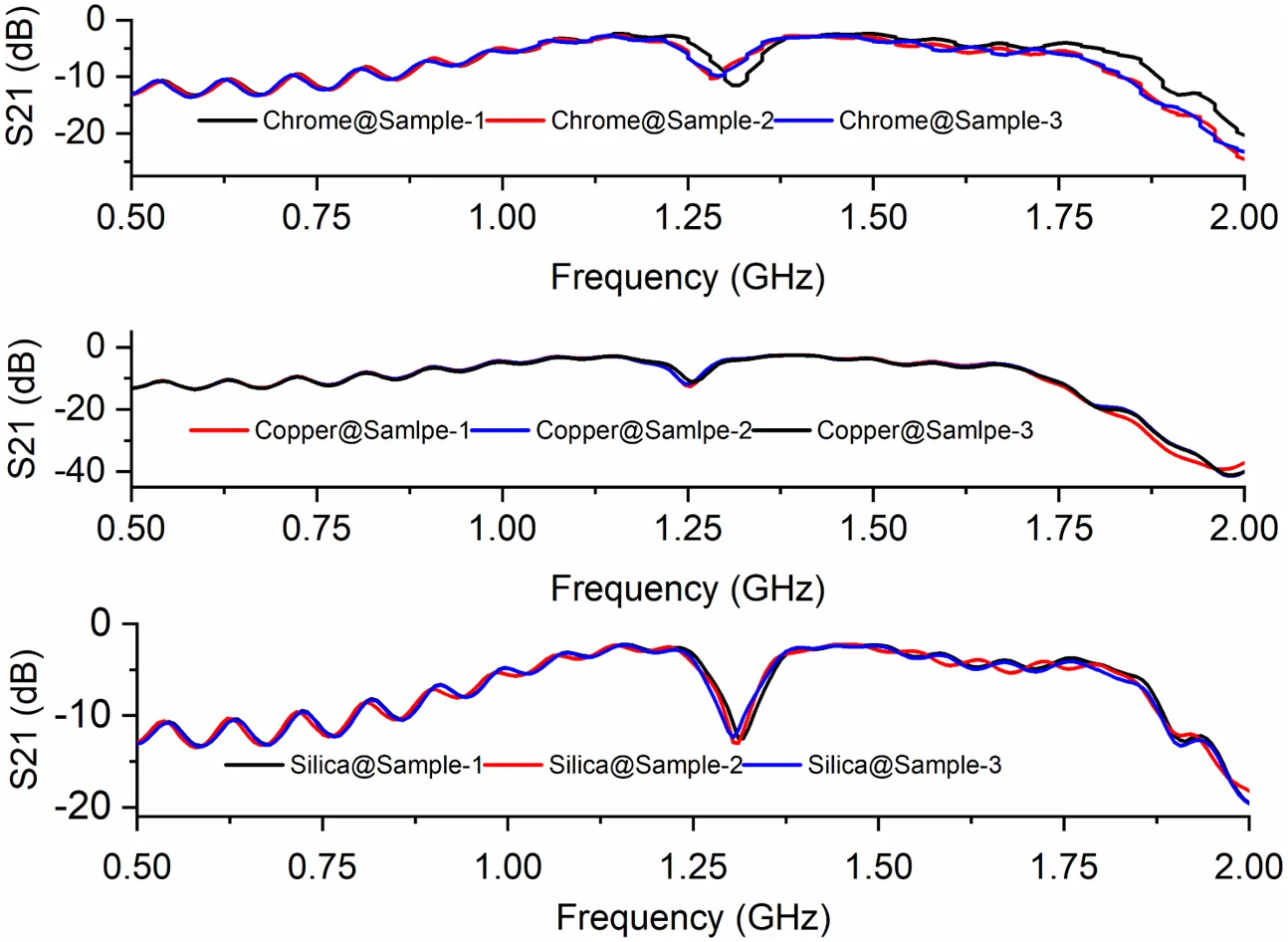
Experimental results of transmission coefficient (S21) for mining-based materials.


$$\begin{array}{c} Sensetivity(S)=\frac{\left(f_{unload}-f_{loaded}\right)}{f_{unload}\left(\varepsilon_{r}-\text{1}\right)}\times \text{1}\text{0}\text{0} \end{array}$$ (2)

Where, $f_{unload}$ is resonating frequency of the sensor without loading sample, $f_{loaded}$ is resonating frequency of the sensor loaded with sample and $\varepsilon_{r}$ relative permittivity of the tested sample. The sensitivities for each category (4g,8g,12g) sample of chrome, copper and silica are shown in Fig 9. The sensitivity for chrome is (1.94,2.4,2.32), the copper samples have sensitivity (4.35,4.56,4.14) and sensitivity for silica is (5.27,5.6,5.6) for (4g,8g,12g) respectively. The maximum sensitivity was identified as the silica sample.

**Fig 9:**
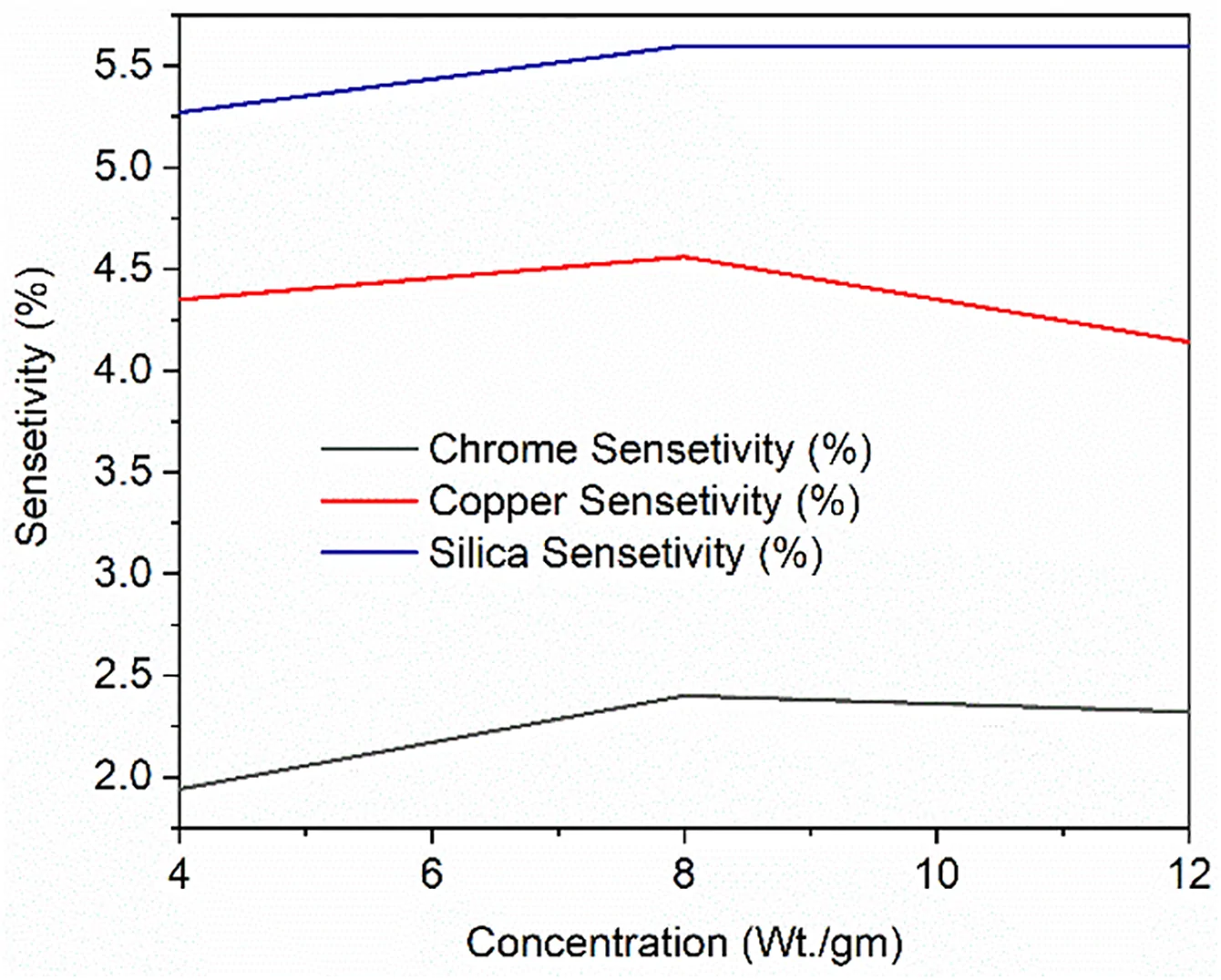
Sensitivity of the proposed sensor for different samples.

## 4. Second version of the proposed sensor

The sensor dimensions are optimized using the CST optimizer, resulting in a compact structure of 20 × 30 mm^2^ with an operating frequency range of 4–6 GHz. This optimized version is designed and fabricated on the Rogers RT5870 substrate having dielectric constant 2.33 and height 0.8 mm. Simulations are conducted with and without a circular sample holder (height: 10 mm, diameter: 18 mm). As shown in Fig 10, the sensor exhibits a resonance at 4.88 GHz with (S21) minimum value of –42.38 dB for measured result. The fabricated prototype is further validated through comparing the measured and simulated results.

**Fig 10:**
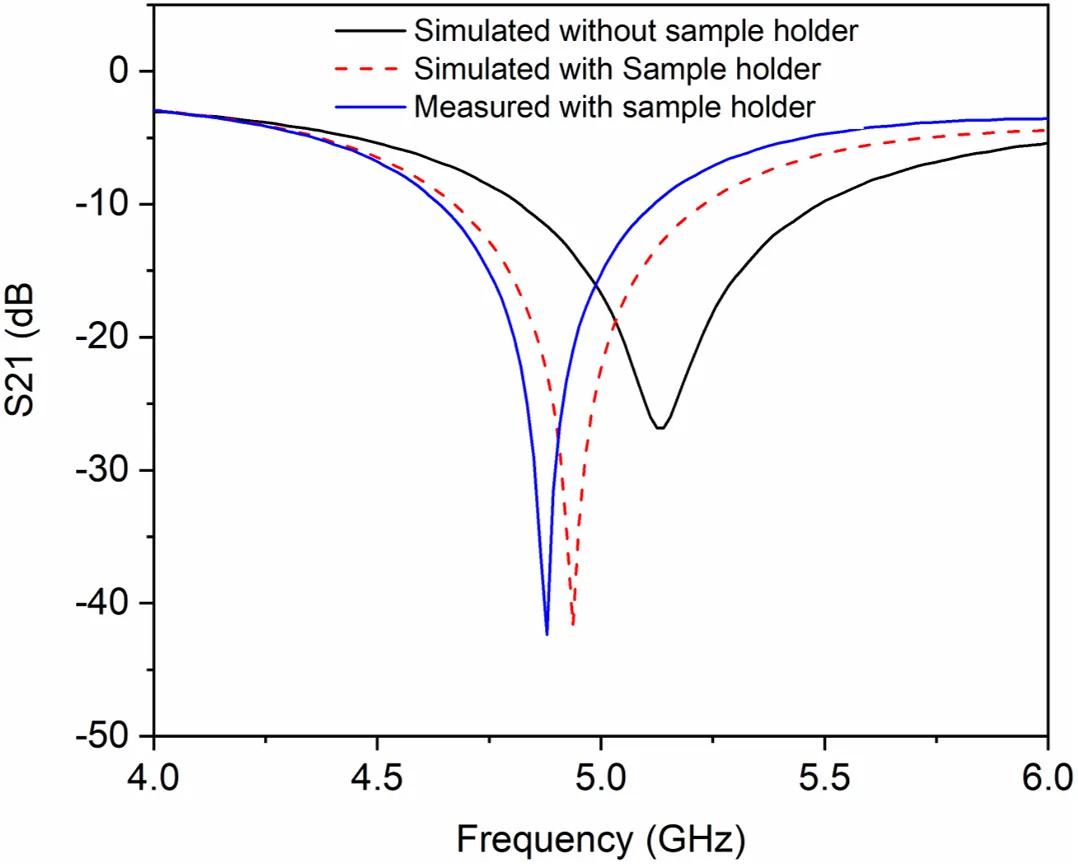
Simulated and measured transmission coefficient (S21) results of optimized sensor.

Sample analysis was performed for all three types of minerals using the optimized version of the sensor. In this measurement, all samples (4g,8g and 12g) were used as prepared in the previous section. The transmission coefficient (S21) results for each sample are depicted in Fig 11, exhibiting a clear resonance shift for the copper, chrome, and silica samples.

**Fig 11:**
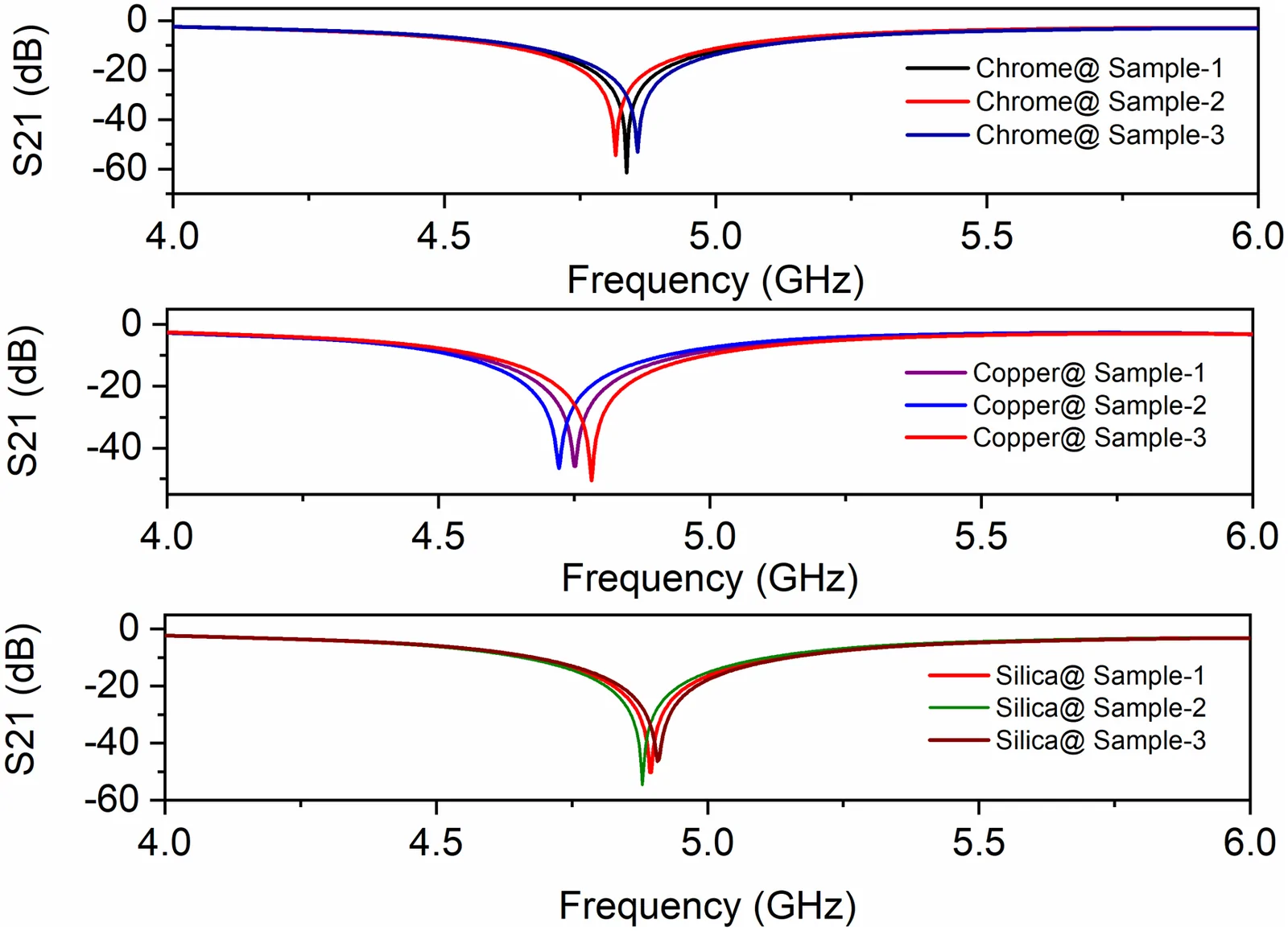
Experimental results of transmission coefficient (S21) for mining-based materials using optimized second version of the sensor.

The resonate frequency, minimum value of (S21), quality factor (Q), sensitivity, and Figure of Merit (FOM) for chrome, copper and silica samples are summarized in Table 3–5 respectively. The results show that all chrome samples maintain a stable resonant response with a narrow 0.004 GHz bandwidth, producing highest (Q) factor for sample-3, i.e., (1214). While Sample-1 serves as the reference, Sample-2 and Sample −3 exhibit small but clear resonant frequency shifts, reflected in their sensitivities of 0.020 GHz and 0.040 GHz. These shifts demonstrate the sensor’s ability to detect incremental changes in the samples. Additionally, the (FOM) increases from 5.00 to 10.00 from Sample-2 to Sample-3, indicating improved sensing performance as the sensitivity rises while the resonance sharpness remains high.

**Table 3:** Key performance parameters for chrome samples.

Sample	Resonant Frequency (GHz)	Min S21 (dB)	Bandwidth (GHz)	Quality Factor (Q)	Sensitivity (GHz)	FOM
Chrome@ Sample-1	4.836	−61.37	0.0040	1209.09	—	—
Chrome@ Sample-2	4.816	−54.31	0.0040	1204.09	0.0200	5.00
Chrome@ Sample-3	4.856	−52.97	0.0040	1214.09	0.0400	10.00

**Table 4:** Key performance parameters for copper samples.

Sample	Resonant Frequency (GHz)	Min S21 (dB)	Bandwidth (GHz)	Quality Factor (Q)	Sensitivity (GHz)	FOM
Copper@ Sample-1	4.752	−45.83	0.0100	475.19	—	—
Copper@ Sample-2	4.722	−46.50	0.0080	590.26	0.0300	3.75
Copper@ Sample-3	4.782	−50.50	0.0060	796.99	0.0600	10.00

**Table 5:** Key performance parameters for silica samples.

Sample	Resonant Frequency (GHz)	Min S21 (dB)	Bandwidth (GHz)	Quality Factor (Q)	Sensitivity (GHz)	FOM
Silica@ Sample-1	4.896	−50.18	0.0060	816.06	—	—
Silica@ Sample-2	4.880	−54.66	0.0040	1219.94	0.0160	4.00
Silica@ Sample-3	4.908	−46.39	0.0100	490.79	0.0280	2.80

The copper samples demonstrate a clear enhancement in sensing performance with increasing concentration, as evidenced by the resonance shift, decreasing bandwidth, and deeper (S21) values. The reduction in bandwidth leads to a higher (Q) factor, indicating sharper and more selective resonance. Simultaneously, sensitivity doubles and the (FOM) increased significantly, confirming improved detection capability.

The silica samples show non-monotonic resonance behavior with concentration, indicating unstable sensor response. Although, bandwidth reduced at Sample-2, yielding a high Q (~1220), it increases significantly at Sample-3, causing Q degradation. The (S21) depth also varies inconsistently, reflecting non-uniform field interaction. While sensitivity increases, the (FOM) decreases at higher concentration due to bandwidth broadening, suggesting reduced sensing efficiency. In conclusion, silica demonstrated optimal performance only at intermediate concentration, with degraded reliability at higher loading.

A summary of the overall performance comparison for all three minerals tested using the optimized sensor is presented in Table 6. The chrome measurements demonstrated the best overall performance, characterized by the highest Q-factor and (FOM), indicating extremely sharp resonance and superior sensing capability. In comparison, copper sample exhibited a strong and consistent progressive behavior, with sensing parameters such as Q-factor, sensitivity, and FOM improving steadily with increasing concentration, reflecting reliable and predictable performance. On the other hand, silica shows comparatively less reliability due to its inconsistent resonance shifts and fluctuating bandwidth, which lead to non-uniform Q-factor and reduced sensing efficiency.

**Table 6:** Overall performance comparison among three minerals results.

Mineral	Q-factor	Best Sensitivity	Best FOM	Sensetivity
Copper	High	High	High	Good
Chrome	**Very High**	High	**Best**	**Excellent**
Silica	Moderate	Low	Low	Poor

## 5. Conclusion

In this work, a dual‑version, dual‑scale CSRR‑loaded microwave sensors were designed, fabricated, and experimentally verified. Both sensor prototypes exhibited distinct and sharply defined resonant frequencies at 1.5 GHz and 4.88 GHz, respectively, enabling multi‑scale characterization within a compact structure. Three mineral samples Copper, Chrome, and Silica were analyzed using each fabricated sensor, demonstrating the sensor’s capability to differentiate materials based on their electromagnetic dielectric contrast. The second version of the sensor for chrome sample analysis is offered high Q-factor (1214) and best Figure of Merit (10). The combined advantages of compactness, low cost, and dual‑scale operation make the proposed sensor a strong candidate for non‑destructive material characterization. Additionally, the methodology presented in this study provides a foundation for developing portable, field‑deployable sensing devices tailored for rapid, on‑site evaluation of mining and mineral‑based materials.
