# Supplementary material for: Dual-version and dual-scale CSRR loaded sensor for mining-based material characterization
Source: PLoS One. 2026 Oct 9;21(10):e0360251. doi: 10.1371/journal.pone.0360251 (PMC13652275; doi:10.1371/journal.pone.0360251)
Supplement: Supplementary file 1 [file pone.0360251.s002.docx]

Submission ID: **PONE-D-25-62986**

**Original Article Title: "** **Dual-Version and Dual-Scale CSRR Loaded Sensor for Mining-Based Material Characterization"**

**To:** Submission ID **PONE-D-25-62986	(Revision-1)**

**Re:** Response to reviewers

Dear Editor-in-Chief,

Thank you for allowing the resubmission of our manuscript, with an opportunity to address the reviewers’ comments.

Best regards,

Madan Kumar Sharma

**Author response:**

We would like to thank the Chief Editor, and Editor of PLOS One journal for having provided the helpful review report, and for having given us a chance for doing revision of the manuscript. Based on comments and suggestions from the reviewers, we have incorporated points as suggested by respected reviewer and editor. We hope the editor and the reviewers will be satisfied with these revisions. Kindly refer to the following sections for these responses.

We are uploading two files, (a) our point-by-point response to the comments (below) (response to reviewers), (b) an updated manuscript with indicating changes in response to all reviewer’s comments with highlighted different colors, (c) Clean copy of the manuscript.

**Few important points regarding revisions are addressed below:**

1. Comments & responses to reviewer #2 are addressed by green font.
2. Comments & responses to reviewer #3 are addressed by blue font.
3. There are some updates about figures and table as mentioned below:

| **Previous** | **Revised** | **Remark** |
| --- | --- | --- |
| Fig.2 | Fig.2 | Revised as per comment#3 of reviwer#2, legend updated. |
| Fig.10 | Fig.10 | Revised as per comment#3 of reviwer#2, legend updated. |
| Fig.11 | Fig.11 | Revised as per comment#7 of reviwer#3 |
| - | Table-2 | Added new as per comment#8 of reviwer#3 |
| - | Table-3 | Added new as per comment#8 of reviwer#3 |
| - | Table-4 | Added new as per comment#8 of reviwer#3 |
| - | Table-5 | Added new as per comment#8 of reviwer#3 |

In addition, following new references are added:

[1] A. M. Lang, K. Aasly, and S. L. Ellefmo, “Mineral characterization as a tool in the implementation of geometallurgy into industrial mineral mining,” Miner. Eng., vol. 116, pp. 114–122, Jan. 2018, doi: 10.1016/j.mineng.2017.10.021.

[2] A. Ali, N. Zhang, and R. M. Santos, “Mineral Characterization Using Scanning Electron Microscopy (SEM): A Review of the Fundamentals, Advancements, and Research Directions,” Applied Sciences 2023, Vol. 13, vol. 13, no. 23, Nov. 2023, doi: 10.3390/app132312600.

[3] N. Javanbakht, G. Xiao, and R. E. Amaya, “A Comprehensive Review of Portable Microwave Sensors for Grains and Mineral Materials Moisture Content Monitoring,” IEEE Access, vol. 9, pp. 120176–120184, 2021, doi: 10.1109/ACCESS.2021.3108906.

======================================================================

**Editor Comments & Response**

===Comments& Response=============================================================

======================================================================

In addition to reviewer comment:

**Comment#1** In the revised manuscript the authors must state the novelty statement to journal standard.

**Response#1** Thank you for your advice this is included in introduction section last paragraph as below:

In this work, a novel dual‑version and dual‑scale CSRR‑loaded sensor is presented. The sensor is specifically engineered to enhance the detection and differentiation of material properties commonly encountered in mining environments. The proposed sensor architecture leverages with CSRR array and slot stubs to achieve improved electromagnetic interaction with heterogeneous mining samples, thereby enabling precise and reliable material characterization.

**Comment#2** The CST simulation code must be deposited into the GitHub account of the correspondent/co-author and the link is provided in the manuscript.

**Response#2** Thank you for your kind suggestion, we have included it in the revised manuscript in data availability statement.

**Reviwer#1**

===Comments& Response=============================================================

======================================================================

I have carefully reviewed the revised manuscript along with the authors’ detailed responses to the previous comments. I am satisfied that all concerns raised in the earlier round of review have been fully and appropriately addressed. The authors have substantially improved the clarity, completeness, and scientific rigor of the manuscript.

The revision adds significant value in terms of technical content, presentation, and reproducibility, and the manuscript now meets the publication standards of the journal.

**Author’s Response** Thank you for your valuable time and comments to assess this manuscript and provide constructive feedback on it.

Authors are grateful to provide a constructive assessment on the submission. We have incorporated

**Reviewer-2**

===Comments& Response=============================================================

The authors investigated a "Dual-Version and Dual-Scale CSRR Loaded Sensor for Mining-Based Material Characterization". The article is well organized with simulation results, equivalent circuit and measured results. However, a few points need to be clarified for the benefits of the readers.

**Author’s Response:** We are thankful to the reviewer for providing valuable guidance and giving us a chance to improve this manuscript. Author will put every effort into improving the manuscript as per your given comments:

Please consider these changes highlighted by green font in the revised manuscript.

**Comment#1** The introduction should be more focused with respect to the title of the article.

**Response#1** Thank you for your valuable feedback. We appreciate your observation and agree that the introduction should more clearly reflect the core focus of the article. In our revised version, we have strengthened the introduction by highlighting the relevance of dual‑version and dual‑scale Complementary Split Ring Resonator (CSRR) structures for mining‑based material characterization. The updated introduction now provides clearer motivation, background context, and a direct link between existing challenges in mining material assessment and the proposed CSRR‑loaded sensor design. Kindly refer to revised manuscript Introduction section Paragraph-1 and 4^th^ paragraph. Revised introduction has included more details around the title and same revision is shown below:

**Para-1:** Accurate characterization of mining-based materials is critical in determining ore quality and ensuring efficient mining processes [1]. However, conventional characterization of mining-based materials is often labor-intensive, expensive, and not suitable for real-time applications [2]. To mitigate these challenges, resonator-based microwave sensor technology has been considered as a potential solution owing to its high sensitivity and non-destructive evaluation capabilities [3]. The microwave sensors provide material characterization by analyzing the interaction of electromagnetic waves with a Material Under Test (MUT) through absorption, reflection, and transmission, which are influenced by permittivity, conductivity, and permeability [4], [5]. Their resonating behavior enhances the sensing accuracy, making them valuable across fields such as agriculture, biomedical, food, construction, and mining. Among various designs, Split Ring Resonators (SRR) and Complementary SRR (CSRR) are widely used for their compact size, high sensitivity, and efficient electromagnetic control.

**Para-4:** Most reported sensors [6], [7], [9], [10], [12], [13], [14], [16]operate with a single resonance, whereas only a few achieve dual [8] or triple-band performance [15]. Similarly, several designs [6], [7], [9], [13], [15], [16]have been validated with liquid samples, whereas others [8], [9], [13], [14] have focused on solids. Only a few studies [17], [18] have addressed the characterization of mining-based substances. This highlights a clear research gap: the need for a versatile sensor capable of dual-scale, dual-version operation, with specific applicability to mining material characterization. In this work, a dual‑version and dual‑scale CSRR‑loaded sensor is presented. The sensor is specifically engineered to enhance the detection and differentiation of material properties commonly encountered in mining environments. The proposed sensor architecture leverages with CSRR array and slot stubs to achieve improved electromagnetic interaction with heterogeneous mining samples, thereby enabling precise and reliable material characterization. The major contributions of this study are outlined as follows:

**Comment#2** In table 1, the rows containing [8] & [9] may be deleted as data are insufficient.

**Response#2** Thank you for your comment. We appreciate your careful review of Table 1. The rows corresponding to references [8] and [9] have been removed, as the available data were indeed insufficient to support meaningful comparison. This revision has now been reflected in the updated version of Table-1and reference numbers updated due to include some more references.

**Comment#3** In Fig 2, it is not clear that the measured result is due to with or without sample holder.

**Response#3** Thank you for your valuable comment. We agree that Fig. 2 required clearer distinction regarding whether the measured results were obtained with or without the sample holder. Legend of the figure updated to make clear difference between these results. This result is measured after final prototype of the sensor with sample holder. Update figures (Fig.2 and Fig.10) are shown below:

**Fig.2.Simulated and measured transmission (S21) coefficient results of the proposed sensor** **with and without sample holder**

**Fig.10. Simulated and measured transmission coefficient (S21) results of optimized sensor**

**Comment#4** The validation of the S21 results due to ECM and CST(Fig 6) are not matched. Justify the reasons of variations.

**Response#4** Thank you for pointing this out. We acknowledge that the S21 responses obtained from the equivalent‑circuit model (ECM) and CST full‑wave simulations in Fig. 6 do not perfectly coincide. The observed variations arise from the following practical and modeling factors:

Modeling abstraction (ECM vs. full‑wave)

The ECM intentionally simplifies distributed electromagnetic effects into lumped elements. As a result, it does not fully capture higher‑order modes, radiation/leakage, mutual coupling beyond nearest elements, and fringing fields that are inherently included in CST. These effects become more pronounced near resonances and at higher frequencies, leading to differences in both magnitude and slight frequency shifts.

Material property assumptions

The ECM assumes frequency‑independent parameters (εr, tan δ, conductor loss) and smooth conductors, while CST uses frequency‑dependent dispersive loss models and can include surface‑roughness and finite conductivity. Small differences in εr/tan δ and metal loss translate into noticeable changes in S21, especially around sharp spectral features.

**Comment#5** It is mentioned that "As shown in Fig. 10, the sensor exhibits a resonance at 5.1 GHz with S11 of –44 dB." But the figure caption says it is S21 plot. Therefore, Fig 10 is confusing.

**Response#5** Thank you for your observation. We agree that Fig. 10 appeared confusing due to the mismatch between the text and the figure caption. The sentence in the manuscript refers to the S11 resonance at 5.1 GHz was wrong by mistake, in actual Figure indicated S21 results. We have revised text and replaced S11 with S21 in text. This data is corrected and update in Section-4 para-1 and same is given below:

As shown in Fig. 10, the sensor exhibits a resonance at 4.88 GHz with (S21) minimum value of –42.38 dB for measured result. The fabricated prototype is further validated through comparing the measured and simulated results.

**Comment#6** What is about plot at Fig 11? It looks like a S11 plot. It should be clarified.

**Response#6** Thank you for your comment. We appreciate your observation regarding Fig. 11. Although the plot may appear similar to an S11 representation, we would like to clarify that the results shown in this figure correspond to the S21 parameter. We have revised this figure for experimented three minerals (chrome, copper and silica) as per reviwer#3, comment#7. Fig. 11 presents S21 data, ensuring there is no confusion for the readers now.

**Comment#7** The conclusion does not reflect the properly about the achievements of this research. It should be modified.

**Response#7** Thank you for your valuable feedback. We appreciate your observation regarding the conclusion section. In response, we have revised the conclusion to more accurately and comprehensively reflect the key achievements of this research. Revised conclusion section is given below:

In this work, a dual‑version, dual‑scale CSRR‑loaded microwave sensors were designed, fabricated, and experimentally verified. Both sensor prototypes exhibited distinct and sharply defined resonant frequencies at 1.5 GHz and 4.88 GHz, respectively, enabling multi‑scale characterization within a compact structure. Three mineral samples Copper, Chrome, and Silica were analyzed using each fabricated sensor, demonstrating the sensor’s capability to differentiate materials based on their electromagnetic dielectric contrast. The second version of the sensor for chrome sample analysis is offered high Q-factor (1214) and best Figure of Merit (10). The combined advantages of compactness, low cost, and dual‑scale operation make the proposed sensor a strong candidate for non‑destructive material characterization. Additionally, the methodology presented in this study provides a foundation for developing portable, field‑deployable sensing devices tailored for rapid, on‑site evaluation of mining and mineral‑based materials.

**Reviewer-3**

===Comments& Response=============================================================

**Comment#1** The manuscript presents the design, fabrication, and experimental validation of a dual-version microwave sensor based on a complementary split-ring resonator (CSRR) array for mining-based material characterization. The sensing structure employs a circular patch loaded with a 3×3 CSRR array combined with rectangular slots to generate resonance in the transmission coefficient. Two versions of the sensor are introduced, operating at approximately 1.5 GHz and 5.1 GHz respectively, with experimental validation performed using copper, chrome, and silica mineral samples. The study claims improved sensing capability through dual-scale resonance and structural compactness.

However, despite the practical implementation and experimental effort, the manuscript contains several fundamental limitations related to novelty, methodological rigor, reproducibility, and scholarly originality, as detailed below.

The proposed sensing principle is based on conventional CSRR/SRR microwave resonator sensing mechanisms, which are widely established for dielectric and material characterization. Previous studies have already demonstrated CSRR-based sensors for solid, liquid, and environmental material detection, including multi-resonance and compact sensing structures. CSRR-based microwave sensing has long been recognized as a standard approach for dielectric characterization using resonance shift mechanisms, which rely on electromagnetic field perturbation caused by material properties (Boybay, M. S., & Ramahi, O. M. (2012). Material characterization using complementary split-ring resonators. IEEE Transactions on instrumentation and Measurement, 61(11), 3039-3046.) The main contribution of the manuscript appears to be geometric scaling and dimensional optimization of known CSRR configurations rather than introducing a new sensing concept, analytical formulation, or algorithmic advancement. The claimed dual-version and dual-scale operation primarily reflects parameter resizing rather than methodological innovation.

**Response#1** Thank you for your thorough and constructive feedback. We agree that CSRR‑based sensing principles are well established, and we appreciate the need to more clearly articulate the novelty of our contribution. While our work builds on conventional CSRR mechanisms, its primary innovation lies in the dual‑version, dual‑scale resonant architecture specifically optimized for mining‑grade solid material characterization, which has not been extensively explored in prior studies. The integration of a 3×3 CSRR array with targeted rectangular slot perturbations enables simultaneous low‑frequency at 1.5 GHz and high‑frequency 5.1 GHz sensitivity, allowing complementary penetration depth and surface‑level resolution beneficial for heterogeneous mineral samples. Additionally, the manuscript introduces a compact, fabrication‑friendly circular patch configuration, experimentally validated on three industrially relevant minerals, demonstrating practical tunability and enhanced sensing range. We have revised the introduction section last paragraph of the manuscript to more explicitly highlight these contributions, strengthen methodological clarity, and better position our study within the existing CSRR literature.

**Comment#2** A major scholarly concern arises from the existence of earlier publications by the same research group that describe highly similar sensing concepts, structures, and applications.

For example, previously published work already reported mineral identification using a composite SRR-based microwave sensor designed for detecting minerals such as copper and chromite in similar geological contexts:

Sharma, M. K., Idries, N. K. S., Alkalbani, A. S., Satyanarayana, D., Rathinam, G., & Sharma, A. (2025). Minerals identification and characterization using a new 3D printed composite-structured split ring resonator microwave sensor. Discover Applied Sciences, 7(7), 757.

Additionally, related book chapter publications describing multi-resonating CSRR-based mining material characterization sensors appear to present substantially similar technical content and research objectives. The sensor structure, sensing mechanism, and mineral characterization experiments reported in those works strongly resemble the current submission:

Sharma, M. K., Idries, N. K. S., Alkalbani, A. S., Degala, S., Rathinam, G., & Sharma, A. (2025, July). A New Composite-Structured CSRR Loaded Multi-resonating Sensor for Mining Based Material Characterization. In International Conference on ICT for Sustainable Development (pp. 84-92). Cham: Springer Nature Switzerland.

**Response#2** Thank you for this observation and for carefully reviewing the prior work from our research group. We acknowledge that the cited publications share thematic continuity with the current submission, as they all fall within our broader research program focused on developing resonator‑based microwave sensors for geological and mining applications. However, we would like to clarify several key distinctions that demonstrate the novelty and substantive advancement of the present study relative to the previously published work.

In the second cited paper: only simulation study of the proposed sensor was discussed by same research group, this study is extension of this conference paper and implemented sensor into physical form and conducted real experiments for mineral characterization.

**Comment#3** The manuscript states that few studies have investigated mining-based material characterization. However, the literature review remains selective and does not provide systematic benchmarking or quantitative comparison showing performance improvement over existing CSRR sensors. The presented comparison table focuses mainly on geometry and operating frequency but omits key sensing performance metrics such as detection limit, measurement uncertainty, and repeatability.

**Response#3** We appreciate the reviewer’s comment. In our revised manuscript, we clarify that, despite an extensive search, there is currently no directly comparable work in the specific area of mining‑based material characterization using CSRR sensors. Existing CSRR studies focus largely on industrial, chemical, or biological materials, and do not address mining applications or similar sensing conditions. For this reason, meaningful quantitative benchmarking using detection limit, repeatability, or uncertainty is not possible across prior studies. Instead, we emphasize the novelty of our work and provide detailed performance evaluation based on our experimental results, establishing a foundation for future comparative research in this emerging domain.

Kindly refer to third paragraph of introduction in revised manuscript for this justification.Same as given below:

Despite a comprehensive search of existing literature, no prior work has been reported in the specific domain of mining‑based material characterization employing CSRR sensors. Indeed, all the existing works on CSRR sensors have been carried out on industrial, chemical, and biological materials. Therefore, there is a limitation to execute quantitative comparison with the existing literature. However, we compared and discussed related literature of the resonating sensor with proposed work in Table-1. Instead, the novelty of the work is emphasized, and the performance evaluation is carried out in detail using the experimental results obtained.

**Comment#4** The sensor design relies heavily on numerical optimization using CST simulation without providing a generalized theoretical or analytical design methodology.

**Response#4** We thank the reviewer for this observation. Numerical optimization in CST is indeed central to CSRR‑based sensor development due to the complexity of electromagnetic behavior, and simulation‑driven refinement is often necessary to achieve high sensitivity and application‑specific performance. We acknowledge the importance of generalized analytical design methodologies, and this feedback is valuable for guiding future work toward broader theoretical frameworks.

**Comment#5** The equivalent circuit model is presented but lacks parameter extraction validation or quantitative error comparison between circuit model and electromagnetic simulation.

**Response#5** We thank the reviewer for this valuable observation. The equivalent circuit model in our work is primarily intended to offer a conceptual understanding of the sensor’s resonance behavior rather than to serve as a fully validated predictive model. We acknowledge that quantitative parameter extraction and an explicit error comparison with electromagnetic simulations would strengthen the analytical rigor.

**Comment#6** Only three mineral materials are tested, which is insufficient to demonstrate general sensing applicability.

**Response#6** We thank the reviewer for this comment. The selection of copper, chrome, and silica was intentional, as these materials represent key mineral resources of relevance to the Omani mining sector and therefore align directly with the intended application domain of the proposed sensor. Our objective was to evaluate sensor behavior using representative materials rather than to demonstrate universal applicability across all mineral types. We agree that testing a broader range of materials could further establish generalizability, and we consider this an important direction for future work building on the current application‑focused study.

**Comment#7** The second optimized sensor version is validated using only one concentration level, weakening the claimed dual-scale sensing reliability.

**Response#7** Thank you for highlighting this important point. We agree that validating the second optimized sensor version using a single concentration level could limit the perceived robustness of the dual‑scale sensing performance. In response, we have expanded the validation experiments by incorporating multiple concentration levels. These additional measurements are now included in the revised manuscript (see Section 4 and Figure 11), demonstrating consistent sensor behavior across the full targeted detection range. This extended dataset strengthens the reliability of the dual-scale sensing claim and provides a more comprehensive evaluation of the optimized sensor’s performance. Revised Fig.11 is shown below:

**Fig.11. Experimental results of transmission coefficient (S21) for mining-based materials using optimized second version of the sensor**

**Comment#8** The results section mainly describes resonance shifts without providing deeper electromagnetic or physical interpretation.

**Response#8** We thank the reviewer for the comment. The electromagnetic behavior and resonating principle of the sensor, including the electric‑field distribution analysis and the resonance‑shift formulation in Equation (1), are already explained in Section 2.1. However, to strengthen the discussion paragraph of optimized sensor (Section 4), we have now added sample analysis based on the quality factor, figure of merit, sensitivity. For this revision we included new tables (Table-2, Table-3, Table-4) for each mineral and same are given below:

The resonate frequency, minimum value of (S21), quality factor (Q), sensitivity, and Figure of Merit (FOM) for chrome, copper and silica samples are summarized in Table-2, Table-3, and Table-4 respectively. The results show that all chrome samples maintain a stable resonant response with a narrow 0.004 GHz bandwidth, producing highest (Q) factor for sample-3 i.e. (1214). While Sample‑1 serves as the reference, Sample‑2 and Sample ‑3 exhibit small but clear resonant frequency shifts, reflected in their sensitivities of 0.020 GHz and 0.040 GHz. These shifts demonstrate the sensor’s ability to detect incremental changes in the samples. Additionally, the (FOM) increases from 5.00 to 10.00 from Sample‑2 to Sample‑3, indicating improved sensing performance as the sensitivity rises while the resonance sharpness remains high.

**Table-2 Key performance parameters for chrome samples**

| **Sample** | **Resonant Frequency (GHz)** | **Min S21 (dB)** | **Bandwidth (GHz)** | **Quality Factor (Q)** | **Sensitivity (GHz)** | **FOM** |
| --- | --- | --- | --- | --- | --- | --- |
| Chrome@ Sample-1 | 4.836 | -61.37 | 0.0040 | 1209.09 | — | — |
| Chrome@ Sample-2 | 4.816 | -54.31 | 0.0040 | 1204.09 | 0.0200 | 5.00 |
| Chrome@ Sample-3 | 4.856 | -52.97 | 0.0040 | 1214.09 | 0.0400 | 10.00 |

The copper samples demonstrate a clear enhancement in sensing performance with increasing concentration, as evidenced by the resonance shift, decreasing bandwidth, and deeper (S21) values. The reduction in bandwidth leads to a higher (Q) factor, indicating sharper and more selective resonance. Simultaneously, sensitivity doubles and the (FOM) increased significantly, confirming improved detection capability.

**Table-3 Key performance parameters for copper samples**

| **Sample** | **Resonant Frequency (GHz)** | **Min S21 (dB)** | **Bandwidth (GHz)** | **Quality Factor (Q)** | **Sensitivity (GHz)** | **FOM** |
| --- | --- | --- | --- | --- | --- | --- |
| Copper@ Sample-1 | 4.752 | -45.83 | 0.0100 | 475.19 | — | — |
| Copper@ Sample-2 | 4.722 | -46.50 | 0.0080 | 590.26 | 0.0300 | 3.75 |
| Copper@ Sample-3 | 4.782 | -50.50 | 0.0060 | 796.99 | 0.0600 | 10.00 |

The silica samples show non-monotonic resonance behavior with concentration, indicating unstable sensor response. Although, bandwidth reduced at Sample-2, yielding a high Q (~1220), it increases significantly at Sample-3, causing Q degradation. The (S21) depth also varies inconsistently, reflecting non-uniform field interaction. While sensitivity increases, the (FOM) decreases at higher concentration due to bandwidth broadening, suggesting reduced sensing efficiency. In conclusion, silica demonstrated optimal performance only at intermediate concentration, with degraded reliability at higher loading.

**Table-4 Key performance parameters for silica samples**

| **Sample** | **Resonant Frequency (GHz)** | **Min S21 (dB)** | **Bandwidth (GHz)** | **Quality Factor (Q)** | **Sensitivity (GHz)** | **FOM** |
| --- | --- | --- | --- | --- | --- | --- |
| Silica@ Sample-1 | 4.896 | -50.18 | 0.0060 | 816.06 | — | — |
| Silica@ Sample-2 | 4.880 | -54.66 | 0.0040 | 1219.94 | 0.0160 | 4.00 |
| Silica@ Sample-3 | 4.908 | -46.39 | 0.0100 | 490.79 | 0.0280 | 2.80 |

A summary of the overall performance comparison for all three minerals tested using the optimized sensor is presented in Table‑5. The chrome measurements demonstrated the best overall performance, characterized by the highest Q-factor and (FOM), indicating extremely sharp resonance and superior sensing capability. In comparison, copper sample exhibited a strong and consistent progressive behavior, with sensing parameters such as Q-factor, sensitivity, and FOM improving steadily with increasing concentration, reflecting reliable and predictable performance. On the other hand, silica shows comparatively less reliability due to its inconsistent resonance shifts and fluctuating bandwidth, which lead to non-uniform Q-factor and reduced sensing efficiency.

**Table-5 Overall performance comparison among three minerals results**

| **Mineral** | **Q-factor** | **Best Sensitivity** | **Best FOM** | **Sensetivity** |
| --- | --- | --- | --- | --- |
| Copper | High | High | High | Good |
| Chrome | **Very High** | High | **Best** | **Excellent** |
| Silica | Moderate | Low | Low | Poor |

=====================================================================

End of responses

Regards

Madan Kumar Sharma

Corresponding author

Madan.k@uob.edu.om

madansharma12@gmail.com
