# Supplementary material for: Dual-version and dual-scale CSRR loaded sensor for mining-based material characterization
Source: PLoS One. 2026 Oct 9;21(10):e0360251. doi: 10.1371/journal.pone.0360251 (PMC13652275; doi:10.1371/journal.pone.0360251)
Supplement: Supplementary file 2 [file pone.0360251.s003.docx]

Submission ID: **PONE-D-25-62986**

**Original Article Title: "** **Dual-Version and Dual-Scale CSRR Loaded Sensor for Mining-Based Material Characterization"**

**To:** Submission ID **PONE-D-25-62986	(Revision-2)**

**Re:** Response to reviewers

Dear Editor-in-Chief,

Thank you for allowing the resubmission of our manuscript, with an opportunity to address the reviewers’ comments.

Best regards,

Madan Kumar Sharma

**Author response:**

We would like to thank the Chief Editor, and Editor of PLOS One journal for having provided the helpful review report, and for having given us a chance for doing revision of the manuscript. Based on comments and suggestions from the reviewers, we have incorporated points as suggested by respected reviewer and editor. We hope the editor and the reviewers will be satisfied with these revisions. Kindly refer to the following sections for these responses.

We are uploading two files, (a) our point-by-point response to the comments (below) (response to reviewers), (b) an updated manuscript with indicating changes in response to all reviewer’s comments with highlighted different colors, (c) Clean copy of the manuscript.

**Few important points regarding revisions are addressed below:**

1. Comments & responses to Editor are addressed by green color font.
2. Comments & responses to reviewer #3 are addressed by blue color font.
3. Updated tables and table numbers as below:

| **Previous** | **Revised** | **Remark** |
| --- | --- | --- |
| Table-1 | - | As it is |
|  | Table-2 | Added new table on recommendation of reviewer comment. |
| Table-2 to Table-5 | Table-3 to Table-6 | Only order is updated. |

======================================================================

**Editor Comments & Response**

===Comments& Response=============================================================

======================================================================

In addition to reviewer comment:

**Comment#1**

How were variations in sample particle size, packing density, moisture content, and sample placement controlled to ensure reproducible sensing responses?

**Response#1**

Thanks for raising this very important point. In order to obtain reproducible responses from the sensors, all the samples were prepared and tested under the same conditions. The particle size distribution was kept constant by sieving the sample through the same mesh size before carrying out the measurements. The packing density was ensured by keeping the mass of the sample as well as the loading method same for all the tests. Variations due to moisture content were taken care of by drying the samples under controlled conditions before carrying out the measurements and then storing them in a desiccator. Moreover, the samples were placed in the same place inside the sensing chamber.

**Comment#2**

What is the specific advantage of using both scales compared with a single optimized sensor for mineral characterization?

**Response#2**

Thank you for this insightful comment. The key advantage of the dual-frequency sensor is that it captures dielectric responses at two different frequency scales, providing complementary information that cannot be obtained from a single-frequency sensor. Since mining materials exhibit frequency-dependent dielectric characteristics, the lower-frequency resonance probes bulk properties while the higher-frequency resonance offers greater sensitivity to subtle compositional variations. Consequently, the dual-frequency approach improves material discrimination, dielectric-property estimation accuracy, and overall characterization reliability compared to a single optimized sensor.

**Comment#3**

Please clarify the equations used and explain whether these parameters are directly comparable across different resonant frequencies and sensor dimensions.

**Response#3**

Thank you for this valuable comment. These equations describe the fundamental sensing parameters derived from the resonant response of the microwave sensor and are not restricted to a particular resonance mode or operating frequency. Therefore, Equations (1) and (2) are applicable to each resonant frequency exhibited by the sensor, including both frequency bands considered in this work.

Regarding parameter comparison across different resonant frequencies and sensor dimensions, the extracted quantities can be directly compared only when they are appropriately normalized or interpreted within the context of their corresponding resonance mode. Since resonant frequency, quality factor, and field distribution are inherently dependent on the sensor geometry and operating frequency, the absolute values of the measured responses may vary between different sensor dimensions or resonance bands. Nevertheless, Equations (1) and (2) remain valid for all resonances because they are based on the same underlying perturbation mechanism relating the resonant characteristics to the dielectric properties of the material under test.

**Comment#4**

Have the authors evaluated its performance using real heterogeneous mineral samples containing impurities rather than only selected laboratory-prepared materials?

**Response#4**

Thank you for this important comment. Yes, the proposed sensor was evaluated using real mining-derived mineral samples containing naturally occurring impurities and compositional variations, rather than being limited to idealized laboratory-prepared materials. These samples inherently exhibit heterogeneity in terms of mineral composition, impurity content, particle morphology, and dielectric characteristics, thereby providing a more realistic assessment of sensor performance.

The experimental results demonstrated that the sensor was capable of producing distinct and repeatable responses for the tested samples despite their inherent variability. This indicates that the proposed dual-frequency sensing approach is sufficiently robust to characterize practical mining materials and is not limited to controlled laboratory conditions. We have clarified this aspect in the revised manuscript and provided additional details regarding the origin and characteristics of the tested mineral samples.

**Comment#5**

To enhance the manuscript's impact and appeal to a broader scientific readership, consider incorporating recent references to ensure completeness. These references should be included in the Introduction or other relevant sections as appropriate. Additionally, avoid citing more than five references within a single sentence to maintain clarity and readability.

**Response#5**

We sincerely thank the reviewer for this valuable suggestion. In response, we have carefully reviewed the recent literature and incorporated several up-to-date and relevant references throughout the manuscript, particularly in the Introduction and discussion sections, to improve the completeness and broader scientific context of our study. Furthermore, we have revised the citation style to avoid citing more than five references within a single sentence. Where necessary, references have been redistributed across multiple sentences to enhance clarity, readability, and overall presentation of the manuscript. We believe these revisions have strengthened the manuscript and improved its accessibility to a wider scientific audience. Newly latest references are given below:

[6]	M. K. Sharma, A. S. Alkalbani, S. B. M. Sulaiman, and S. Degala, “Low-cost disposable HSRR-biosensor for skin disease characterization and deep-learning assist decisions,” *Journal of Hazardous Materials Advances*, vol. 23, p. 101327, Aug. 2026, doi: 10.1016/J.HAZADV.2026.101327.

[7]	W. Liu and Z. Su, “Multi-band microwave sensor for agricultural microsample analysis using dual asymmetric metamaterial resonators toward enhanced and balanced full-band sensitivity,” *Comput. Electron. Agric.*, vol. 250, p. 111839, Aug. 2026, doi: 10.1016/J.COMPAG.2026.111839.

[8]	M. A. Aldhaeebi, A. Ali, and T. S. Almoneef, “A Machine Learning-Augmented Microwave Sensor for Metallic Landmine Detection,” *Signals 2026, Vol. 7, Page 40*, vol. 7, no. 3, p. 40, May 2026, doi: 10.3390/SIGNALS7030040.

**Comment#6** Authors are encouraged to write the sentences below in the introduction section, incorporating the citations to enhance broader appeal and readability.

**Response#6** Thank you for your kind suggestion. As written text in comment letter has been incorporated revised manuscript and same given below including recommended citations.

In order to provide dependable material characterization and monitoring, recent developments in sensor technology have shown the significance of multi-sensor dependency modeling, flexible sensing platforms, wireless sensor designs, and enhanced calibration techniques [20], [21], [22]. Multi-scale sensing approaches are becoming increasingly important, as evidenced by the development of high-precision cross-scale characterization techniques for complicated three-dimensional objects [23]. Additionally, sensor array accuracy and reliability have been enhanced by directionally regularized calibration techniques and physics-inspired error modeling [24] . The adaptability of modern sensing systems for high-frequency and condition-monitoring applications is further demonstrated by recent advancements in time-frequency integration architectures and planar magnetically linked current sensors[25], [26] . In mining-related settings, image-processing-based techniques have also been effectively used for material identification and classification [27]. Furthermore, guided-surface lattice resonances and high-Q resonant structures have garnered significant interest because of their acute resonance features and increased sensitivity [28]. For cutting-edge industrial applications, sustainable material-based sensing and prediction techniques are also developed in [29]. The creation of the suggested dual-version and dual-scale CSRR-loaded sensor for mining-based material characterization is supported by the collective findings of this research, which demonstrate the increasing demand for small, precise, high-Q, and multi-scale sensing platforms.

**Reviwer#2**

===Comments& Response=================================================================

**Comment:** All comments have been addressed.
**Author’s Response** Thank you for your valuable time and reviewing this manuscript and provide your constructive feedback on it.

**Reviewer-3**

===Comments& Response===================================================================

When comparing the revised manuscript with the previous version, the authors have improved the presentation by revising the introduction, figures, conclusion, and adding some performance metrics. However, the major concerns remain insufficiently addressed.

**Author’s Response:**

We sincerely thank the reviewer for acknowledging the improvements made in the revised manuscript. We also appreciate the observation that some of the major concerns were not sufficiently addressed in the previous revision. In response, we have carefully re-examined all comments and made substantial additional revisions to strengthen the manuscript.

**Comment#1**

The most critical unresolved issue is the relationship between the present manuscript and the authors’ previously published conference/book chapter paper: Sharma et al., “A New Composite-Structured CSRR Loaded Multi-resonating Sensor for Mining Based Material Characterization,” ICT4SD 2025, Springer. The authors acknowledge that the earlier paper presented the same proposed sensor concept and that the current manuscript is an extension involving fabrication and experimental validation. However, no detailed comparison is provided to clearly distinguish previously published material from genuinely new scientific content. Therefore, concerns regarding substantial overlap, incremental publication, and insufficient originality remain unresolved.

**Response#1**

We sincerely thank the reviewer for highlighting this important concern regarding the relationship between the current manuscript and our previously published conference/book chapter paper (Sharma et al., ICT4SD 2025, Springer). We fully agree that a clear distinction between the previously published work and the novel contribution of the present manuscript is essential to avoid any ambiguity regarding originality and scientific contribution.
In contrast, the present manuscript substantially extends that work through several new contributions, including:

**1.Fabrication and Experimental Validation:** The sensor has been physically fabricated and experimentally characterized, which was not included in the conference/book chapter publication.

**2.Comprehensive Measurement-Based Analysis:** Detailed experimental investigations have been carried out to validate the sensing performance under practical conditions, including comparison between simulated and measured results.

**3.Extended Material Characterization Study:** The present work demonstrates the applicability of the proposed sensor for characterization of mining-related materials using real measurements, providing insights that were not available in the previous publication.

**4.Additional Performance Evaluation:** New performance metrics, sensitivity analysis, and validation results have been incorporated to establish the robustness and practical effectiveness of the proposed sensor.

**5.Significantly Expanded Discussion and Interpretation:** The manuscript includes a more comprehensive analysis of the sensing mechanism, experimental observations, limitations, and potential real-world applications.
To ensure transparency, we have added a clear comparison table (Table 2) in the revised manuscript summarizing the scope, methodology, and contributions of both publications. We have also revised the introduction and related sections to explicitly acknowledge the prior work and clearly state the novel scientific contributions presented in the current study.

**Comment#2** In addition, the novelty remains limited, as the work is mainly based on well-established CSRR/SRR microwave sensing principles. The experimental validation is also still limited, with insufficient repeatability, uncertainty analysis, statistical evaluation, and quantitative comparison with existing methods.

**Response#3**

We sincerely thank the reviewer for this insightful comment. We acknowledge that CSRR/SRR-based microwave sensing is a well-established research area. However, the novelty of the present work lies not merely in the use of the CSRR/SRR sensing principle, but in the proposed sensor configuration, its optimization strategy, and its application methodology, which together provide improved sensing performance compared with conventional designs. However, quantitative analysis is done in terms of quality factor (Q-factor), sensitivity, FOM. We included Q-factor in the revised comparison table-1, compared with existing methods. Other quantitative parameters are not completely available in the literature so that unable to include in this comparative study.

We believe that these revisions substantially strengthen the manuscript by providing a clearer demonstration of its novelty, improving the rigor of the experimental validation, and enhancing the overall scientific contribution of the work.

=====================================================================

End of responses

Regards

Madan Kumar Sharma

Corresponding author

Madan.k@uob.edu.om

madansharma12@gmail.com
