# Supplementary material for: Dual-version and dual-scale CSRR loaded sensor for mining-based material characterization
Source: PLoS One. 2026 Oct 9;21(10):e0360251. doi: 10.1371/journal.pone.0360251 (PMC13652275; doi:10.1371/journal.pone.0360251)
Supplement: Peer review history

# Peer review: Decision Letter 0

**DOI:** 10.1371/journal.pone.0360251.r001

**Authors:** Alemayehu Getahun Kumela

12 Feb 2026

Dear Dr. MADAN KUMAR SHARMA,

Thank you for submitting your manuscript to PLOS ONE. After careful consideration, we feel that it has merit but does not fully meet PLOS ONE’s publication criteria as it currently stands. Therefore, we invite you to submit a revised version of the manuscript that addresses the points raised during the review process.

Please submit your revised manuscript by Mar 29 2026 11:59PM. If you will need more time than this to complete your revisions, please reply to this message or contact the journal office at plosone@plos.org. When you're ready to submit your revision, log on to https://www.editorialmanager.com/pone/ and select the 'Submissions Needing Revision' folder to locate your manuscript file.

- A letter that responds to each point raised by the academic editor and reviewer(s). You should upload this letter as a separate file labeled 'Response to Reviewers'.
- A marked-up copy of your manuscript that highlights changes made to the original version. You should upload this as a separate file labeled 'Revised Manuscript with Track Changes'.
- An unmarked version of your revised paper without tracked changes. You should upload this as a separate file labeled 'Manuscript'.

If you would like to make changes to your financial disclosure, please include your updated statement in your cover letter. Guidelines for resubmitting your figure files are available below the reviewer comments at the end of this letter.

If applicable, we recommend that you deposit your laboratory protocols in protocols.io to enhance the reproducibility of your results. Protocols.io assigns your protocol its own identifier (DOI) so that it can be cited independently in the future. For instructions see: https://journals.plos.org/plosone/s/submission-guidelines#loc-laboratory-protocols. Additionally, PLOS ONE offers an option for publishing peer-reviewed Lab Protocol articles, which describe protocols hosted on protocols.io. Read more information on sharing protocols at https://plos.org/protocols?utm_medium=editorial-email&utm_source=authorletters&utm_campaign=protocols.

We look forward to receiving your revised manuscript.

Kind regards,

Alemayehu Getahun Kumela, Ph.D.

Academic Editor

PLOS One

Journal Requirements:

When submitting your revision, we need you to address these additional requirements.

1. Please ensure that your manuscript meets PLOS ONE's style requirements, including those for file naming. The PLOS ONE style templates can be found at

https://journals.plos.org/plosone/s/file?id=wjVg/PLOSOne_formatting_sample_main_body.pdf and

https://journals.plos.org/plosone/s/file?id=ba62/PLOSOne_formatting_sample_title_authors_affiliations.pdf.

2. Please note that PLOS One has specific guidelines on code sharing for submissions in which author-generated code underpins the findings in the manuscript. In these cases, we expect all author-generated code to be made available without restrictions upon publication of the work. Please review our guidelines at https://journals.plos.org/plosone/s/materials-and-software-sharing#loc-sharing-code and ensure that your code is shared in a way that follows best practice and facilitates reproducibility and reuse.

3. Thank you for stating in your Funding Statement:

“This work was financially supported by the Ministry of Higher Education, Research and Innovation (MOHERI), Oman, under the Block Funding Program (Grant Number: BFP/RGP/EI/23/486).”

Please provide an amended statement that declares *all* the funding or sources of support (whether external or internal to your organization) received during this study, as detailed online in our guide for authors at http://journals.plos.org/plosone/s/submit-now.  Please also include the statement “There was no additional external funding received for this study.” in your updated Funding Statement.

Please include your amended Funding Statement within your cover letter. We will change the online submission form on your behalf.

4. Please provide a complete Data Availability Statement in the submission form, ensuring you include all necessary access information or a reason for why you are unable to make your data freely accessible. If your research concerns only data provided within your submission, please write "All data are in the manuscript and/or supporting information files" as your Data Availability Statement.

5. Please be informed that funding information should not appear in the Acknowledgments section or other areas of your manuscript. We will only publish funding information present in the Funding Statement section of the online submission form. Please remove any funding-related text from the manuscript.

6. If the reviewer comments include a recommendation to cite specific previously published works, please review and evaluate these publications to determine whether they are relevant and should be cited. There is no requirement to cite these works unless the editor has indicated otherwise.

[Note: HTML markup is below. Please do not edit.]

Reviewers' comments:

Reviewer's Responses to Questions

**Comments to the Author**

1. Is the manuscript technically sound, and do the data support the conclusions?

Reviewer #1: Yes

Reviewer #2: Yes

Reviewer #3: No

**********

2. Has the statistical analysis been performed appropriately and rigorously?

Reviewer #1: Yes

Reviewer #2: Yes

Reviewer #3: No

**********

3. Have the authors made all data underlying the findings in their manuscript fully available?

The PLOS Data policy

Reviewer #1: Yes

Reviewer #2: Yes

Reviewer #3: Yes

**********

4. Is the manuscript presented in an intelligible fashion and written in standard English?

Reviewer #1: Yes

Reviewer #2: Yes

Reviewer #3: Yes

**********

Reviewer #1: I have carefully reviewed the revised manuscript along with the authors’ detailed responses to the previous comments. I am satisfied that all concerns raised in the earlier round of review have been fully and appropriately addressed. The authors have substantially improved the clarity, completeness, and scientific rigor of the manuscript.

The revision adds significant value in terms of technical content, presentation, and reproducibility, and the manuscript now meets the publication standards of the journal.

Summary of Improvements

1. Novelty and Contributions Clarified

The introduction now clearly articulates the core contributions, and the authors explicitly distinguish their work from existing literature. The novelty regarding antenna compactness, dual‑band functionality, and comprehensive multi‑phantom SAR assessment is now well defined.

2. Expanded Comparison Table

Table 5 has been meaningfully revised to include antenna size, gain, efficiency, and SAR values. Missing metrics in previous works are transparently marked as “not reported,” improving fairness and completeness of comparison.

3. SAR Evaluation Reproducibility

A new table summarizing phantom models, voxel resolution, solver type, dielectric database, spacing, and power levels now ensures transparency and reproducibility of the SAR study.

4. Experimental Section Strengthened

While additional measurements could not be performed due to equipment maintenance, the authors have sufficiently expanded the measurement description and clarified uncertainty sources. The reported results are consistent and reliable within the presented limitations.

5. Figure Quality and Formatting

All figures have been standardized with consistent formatting, labelled axes, units, and SAR color scales. Visual clarity and technical accuracy have been notably improved.

6. Language, Clarity, and Consistency

The manuscript’s writing has been significantly refined for conciseness and better scientific tone. Terminology is unified and quantitative performance metrics are now provided in the Abstract.

7. Responsiveness to Review Comments

The authors provided a thorough, well‑structured, and respectful point‑by‑point response, demonstrating careful consideration of all feedback.

Reviewer #2: The authors investigated a "Dual-Version and Dual-Scale CSRR Loaded Sensor for Mining-Based Material Characterization". The article is well organized with simulation results, equivalent circuit and measured results. However, a few points need to be clarified for the benefits of the readers.

1. The introduction should be more focused with respect to the title of the article.

2. In table 1, the rows containing [8] & [9] may be deleted as data are insufficient.

3. In Fig 2, it is not clear that the measured result is due to with or without sample holder.

4. The validation of the S21 results due to ECM and CST(Fig 6) are not matched. Justify the reasons of variations.

5. It is mentioned that "As shown in Fig. 10, the sensor exhibits a resonance at 5.1 GHz with S11 of –44 dB." But the figure caption says it is S21 plot.Therefore, Fig 10 is confusing.

6. What is about plot at Fig 11? It looks like a S11 plot. It should be clarified.

7. The conclusion does not reflect the properly about the achievements of this research. It should be modified.

Reviewer #3: The manuscript presents the design, fabrication, and experimental validation of a dual-version microwave sensor based on a complementary split-ring resonator (CSRR) array for mining-based material characterization. The sensing structure employs a circular patch loaded with a 3×3 CSRR array combined with rectangular slots to generate resonance in the transmission coefficient. Two versions of the sensor are introduced, operating at approximately 1.5 GHz and 5.1 GHz respectively, with experimental validation performed using copper, chrome, and silica mineral samples. The study claims improved sensing capability through dual-scale resonance and structural compactness.

However, despite the practical implementation and experimental effort, the manuscript contains several fundamental limitations related to novelty, methodological rigor, reproducibility, and scholarly originality, as detailed below.

The proposed sensing principle is based on conventional CSRR/SRR microwave resonator sensing mechanisms, which are widely established for dielectric and material characterization. Previous studies have already demonstrated CSRR-based sensors for solid, liquid, and environmental material detection, including multi-resonance and compact sensing structures. CSRR-based microwave sensing has long been recognized as a standard approach for dielectric characterization using resonance shift mechanisms, which rely on electromagnetic field perturbation caused by material properties (Boybay, M. S., & Ramahi, O. M. (2012). Material characterization using complementary split-ring resonators. IEEE Transactions on instrumentation and Measurement, 61(11), 3039-3046.) The main contribution of the manuscript appears to be geometric scaling and dimensional optimization of known CSRR configurations rather than introducing a new sensing concept, analytical formulation, or algorithmic advancement. The claimed dual-version and dual-scale operation primarily reflects parameter resizing rather than methodological innovation.

A major scholarly concern arises from the existence of earlier publications by the same research group that describe highly similar sensing concepts, structures, and applications.

For example, previously published work already reported mineral identification using a composite SRR-based microwave sensor designed for detecting minerals such as copper and chromite in similar geological contexts:

Sharma, M. K., Idries, N. K. S., Alkalbani, A. S., Satyanarayana, D., Rathinam, G., & Sharma, A. (2025). Minerals identification and characterization using a new 3D printed composite-structured split ring resonator microwave sensor. Discover Applied Sciences, 7(7), 757.

Additionally, related book chapter publications describing multi-resonating CSRR-based mining material characterization sensors appear to present substantially similar technical content and research objectives. The sensor structure, sensing mechanism, and mineral characterization experiments reported in those works strongly resemble the current submission:

Sharma, M. K., Idries, N. K. S., Alkalbani, A. S., Degala, S., Rathinam, G., & Sharma, A. (2025, July). A New Composite-Structured CSRR Loaded Multi-resonating Sensor for Mining Based Material Characterization. In International Conference on ICT for Sustainable Development (pp. 84-92). Cham: Springer Nature Switzerland.

The manuscript states that few studies have investigated mining-based material characterization. However, the literature review remains selective and does not provide systematic benchmarking or quantitative comparison showing performance improvement over existing CSRR sensors. The presented comparison table focuses mainly on geometry and operating frequency but omits key sensing performance metrics such as detection limit, measurement uncertainty, and repeatability.

The sensor design relies heavily on numerical optimization using CST simulation without providing a generalized theoretical or analytical design methodology.

The equivalent circuit model is presented but lacks parameter extraction validation or quantitative error comparison between circuit model and electromagnetic simulation.

Only three mineral materials are tested, which is insufficient to demonstrate general sensing applicability.

The second optimized sensor version is validated using only one concentration level, weakening the claimed dual-scale sensing reliability.

The results section mainly describes resonance shifts without providing deeper electromagnetic or physical interpretation.

**********

what does this mean?). If published, this will include your full peer review and any attached files.

If you choose “no”, your identity will remain anonymous but your review may still be made public.

**Do you want your identity to be public for this peer review?** For information about this choice, including consent withdrawal, please see our Privacy Policy

Reviewer #1: **Yes:**  Esraa Mousa Ali

Reviewer #2: **Yes:**  Dr Santanu Kumar Behera

Reviewer #3: No

**********

[NOTE: If reviewer comments were submitted as an attachment file, they will be attached to this email and accessible via the submission site. Please log into your account, locate the manuscript record, and check for the action link "View Attachments". If this link does not appear, there are no attachment files.]

To ensure your figures meet our technical requirements, please review our figure guidelines: https://journals.plos.org/plosone/s/figures

You may also use PLOS’s free figure tool, NAAS, to help you prepare publication quality figures: https://journals.plos.org/plosone/s/figures#loc-tools-for-figure-preparation.

NAAS will assess whether your figures meet our technical requirements by comparing each figure against our figure specifications.

# Author comment: Author response to Decision Letter 1

**DOI:** 10.1371/journal.pone.0360251.r002

27 Mar 2026

Submission ID: PONE-D-25-62986

Original Article Title: " Dual-Version and Dual-Scale CSRR Loaded Sensor for Mining-Based Material Characterization"

To: Submission ID PONE-D-25-62986 (Revision-1)

Re: Response to reviewers

Dear Editor-in-Chief,

Thank you for allowing the resubmission of our manuscript, with an opportunity to address the reviewers’ comments.

Best regards,

Madan Kumar Sharma

Author response:

We would like to thank the Chief Editor, and Editor of PLOS One journal for having provided the helpful review report, and for having given us a chance for doing revision of the manuscript. Based on comments and suggestions from the reviewers, we have incorporated points as suggested by respected reviewer and editor. We hope the editor and the reviewers will be satisfied with these revisions. Kindly refer to the following sections for these responses.

We are uploading two files, (a) our point-by-point response to the comments (below) (response to reviewers), (b) an updated manuscript with indicating changes in response to all reviewer’s comments with highlighted different colors, (c) Clean copy of the manuscript.

Few important points regarding revisions are addressed below:

1. Comments & responses to reviewer #2 are addressed by green font.

2. Comments & responses to reviewer #3 are addressed by blue font.

3. There are some updates about figures and table as mentioned below:

Previous Revised Remark

Fig.2 Fig.2 Revised as per comment#3 of reviwer#2, legend updated.

Fig.10 Fig.10 Revised as per comment#3 of reviwer#2, legend updated.

Fig.11 Fig.11 Revised as per comment#7 of reviwer#3

- Table-2 Added new as per comment#8 of reviwer#3

- Table-3 Added new as per comment#8 of reviwer#3

- Table-4 Added new as per comment#8 of reviwer#3

- Table-5 Added new as per comment#8 of reviwer#3

In addition, following new references are added:

[1] A. M. Lang, K. Aasly, and S. L. Ellefmo, “Mineral characterization as a tool in the implementation of geometallurgy into industrial mineral mining,” Miner. Eng., vol. 116, pp. 114–122, Jan. 2018, doi: 10.1016/j.mineng.2017.10.021.

[2] A. Ali, N. Zhang, and R. M. Santos, “Mineral Characterization Using Scanning Electron Microscopy (SEM): A Review of the Fundamentals, Advancements, and Research Directions,” Applied Sciences 2023, Vol. 13, vol. 13, no. 23, Nov. 2023, doi: 10.3390/app132312600.

[3] N. Javanbakht, G. Xiao, and R. E. Amaya, “A Comprehensive Review of Portable Microwave Sensors for Grains and Mineral Materials Moisture Content Monitoring,” IEEE Access, vol. 9, pp. 120176–120184, 2021, doi: 10.1109/ACCESS.2021.3108906.

======================================================================

Editor Comments & Response

===Comments& Response=============================================================

======================================================================

In addition to reviewer comment:

Comment#1 In the revised manuscript the authors must state the novelty statement to journal standard.

Response#1 Thank you for your advice this is included in introduction section last paragraph as below:

In this work, a novel dual‑version and dual‑scale CSRR‑loaded sensor is presented. The sensor is specifically engineered to enhance the detection and differentiation of material properties commonly encountered in mining environments. The proposed sensor architecture leverages with CSRR array and slot stubs to achieve improved electromagnetic interaction with heterogeneous mining samples, thereby enabling precise and reliable material characterization.

Comment#2 The CST simulation code must be deposited into the GitHub account of the correspondent/co-author and the link is provided in the manuscript.

Response#2 Thank you for your kind suggestion, we have included it in the revised manuscript in data availability statement.

Reviwer#1

===Comments& Response=============================================================

======================================================================

I have carefully reviewed the revised manuscript along with the authors’ detailed responses to the previous comments. I am satisfied that all concerns raised in the earlier round of review have been fully and appropriately addressed. The authors have substantially improved the clarity, completeness, and scientific rigor of the manuscript.

The revision adds significant value in terms of technical content, presentation, and reproducibility, and the manuscript now meets the publication standards of the journal.

Author’s Response Thank you for your valuable time and comments to assess this manuscript and provide constructive feedback on it.

Authors are grateful to provide a constructive assessment on the submission. We have incorporated

Reviewer-2

===Comments& Response=============================================================

The authors investigated a "Dual-Version and Dual-Scale CSRR Loaded Sensor for Mining-Based Material Characterization". The article is well organized with simulation results, equivalent circuit and measured results. However, a few points need to be clarified for the benefits of the readers.

Author’s Response: We are thankful to the reviewer for providing valuable guidance and giving us a chance to improve this manuscript. Author will put every effort into improving the manuscript as per your given comments:

Please consider these changes highlighted by green font in the revised manuscript.

Comment#1 The introduction should be more focused with respect to the title of the article.

Response#1 Thank you for your valuable feedback. We appreciate your observation and agree that the introduction should more clearly reflect the core focus of the article. In our revised version, we have strengthened the introduction by highlighting the relevance of dual version and dual scale Complementary Split Ring Resonator (CSRR) structures for mining based material characterization. The updated introduction now provides clearer motivation, background context, and a direct link between existing challenges in mining material assessment and the proposed CSRR loaded sensor design. Kindly refer to revised manuscript Introduction section Paragraph-1 and 4th paragraph. Revised introduction has included more details around the title and same revision is shown below:

Para-1: Accurate characterization of mining-based materials is critical in determining ore quality and ensuring efficient mining processes [1]. However, conventional characterization of mining-based materials is often labor-intensive, expensive, and not suitable for real-time applications [2]. To mitigate these challenges, resonator-based microwave sensor technology has been considered as a potential solution owing to its high sensitivity and non-destructive evaluation capabilities [3]. The microwave sensors provide material characterization by analyzing the interaction of electromagnetic waves with a Material Under Test (MUT) through absorption, reflection, and transmission, which are influenced by permittivity, conductivity, and permeability [4], [5]. Their resonating behavior enhances the sensing accuracy, making them valuable across fields such as agriculture, biomedical, food, construction, and mining. Among various designs, Split Ring Resonators (SRR) and Complementary SRR (CSRR) are widely used for their compact size, high sensitivity, and efficient electromagnetic control.

Para-4: Most reported sensors [6], [7], [9], [10], [12], [13], [14], [16]operate with a single resonance, whereas only a few achieve dual [8] or triple-band performance [15]. Similarly, several designs [6], [7], [9], [13], [15], [16]have been validated with liquid samples, whereas others [8], [9], [13], [14] have focused on solids. Only a few studies [17], [18] have addressed the characterization of mining-based substances. This highlights a clear research gap: the need for a versatile sensor capable of dual-scale, dual-version operation, with specific applicability to mining material characterization. In this work, a dual‑version and dual‑scale CSRR‑loaded sensor is presented. The sensor is specifically engineered to enhance the detection and differentiation of material properties commonly encountered in mining environments. The proposed sensor architecture leverages with CSRR array and slot stubs to achieve improved electromagnetic interaction with heterogeneous mining samples, thereby enabling precise and reliable material characterization. The major contributions of this study are outlined as follows:

Comment#2 In table 1, the rows containing [8] & [9] may be deleted as data are insufficient.

Response#2 Thank you for your comment. We appreciate your careful review of Table 1. The rows corresponding to references [8] and [9] have been removed, as the available data were indeed insufficient to support meaningful comparison. This revision has now been reflected in the updated version of Table-1and reference numbers updated due to include some more references.

Comment#3 In Fig 2, it is not clear that the measured result is due to with or without sample holder.

Response#3 Thank you for your valuable comment. We agree that Fig. 2 required clearer distinction regarding whether the measured results were obtained with or without the sample holder. Legend of the figure updated to make clear difference between these results. This result is measured after final prototype of the sensor with sample holder. Update figures (Fig.2 and Fig.10) are shown below:

Fig.2.Simulated and measured transmission (S21) coefficient results of the proposed sensor with and without sample holder

Fig.10. Simulated and measured transmission coefficient (S21) results of optimized sensor

Comment#4 The validation of the S21 results due to ECM and CST(Fig 6) are not matched. Justify the reasons of variations.

Response#4 Thank you for pointing this out. We acknowledge that the S21 responses obtained from the equivalent‑circuit model (ECM) and CST full‑wave simulations in Fig. 6 do not perfectly coincide. The observed variations arise from the following practical and modeling factors:

Modeling abstraction (ECM vs. full‑wave)

The ECM intentionally simplifies distributed electromagnetic effects into lumped elements. As a result, it does not fully capture higher‑order modes, radiation/leakage, mutual coupling beyond nearest elements, and fringing fields that are inherently included in CST. These effects become more pronounced near resonances and at higher frequencies, leading to differences in both magnitude and slight frequency shifts.

Material property assumptions

The ECM assumes frequency‑independent parameters (εr, tan δ, conductor loss) and smooth conductors, while CST uses frequency‑dependent dispersive loss models and can include surface‑roughness and finite conductivity. Small differences in εr/tan δ and metal loss translate into noticeable changes in S21, especially around sharp spectral features.

Comment#5 It is mentioned that "As shown in Fig. 10, the sensor exhibits a resonance at 5.1 GHz with S11 of –44 dB." But the figure caption says it is S21 plot. Therefore, Fig 10 is confusing.

Response#5 Thank you for your observation. We agree that Fig. 10 appeared confusing due to the mismatch between the text and the figure caption. The sentence in the manuscript refers to the S11 resonance at 5.1 GHz was wrong by mistake, in actual Figure indicated S21 results. We have revised text and replaced S11 with S21 in text. This data is corrected and update in Section-4 para-1 and same is given below:

As shown in Fig. 10, the sensor exhibits a resonance at 4.88 GHz with (S21) minimum value of –42.38 dB for measured result. The fabricated prototype is further validated through comparing the measured and simulated results.

Comment#6 What is about plot at Fig 11? It looks like a S11 plot. It should be clarified.

Response#6 Thank you for your comment. We appreciate your observation regarding Fig. 11. Although the plot may appear similar to an S11 representation, we would like to clarify that the results shown in this figure correspond to the S21 parameter. We have revised this figure for experimented three minerals (chrome, copper and silica) as per reviwer#3, comment#7. Fig. 11 presents S21 data, ensuring there is no confusion for the readers now.

Comment#7 The conclusion does not reflect the properly about the achievements of this research. It should be modified.

Response#7 Thank you for your valuable feedback. We appreciate your observation regarding the conclusion section. In response, we have revised the conclusion to more accurately and comprehensively reflect the key achievements of this research. Revised conclusion section is given below:

In this work, a dual‑version, dual‑scale CSRR‑loaded microwave sensors were designed, fabricated, and experimentally verified. Both sensor prototypes exhibited distinct and sharply defined resonant frequencies at 1.5 GHz and 4.88 GHz, respectively, enabling multi‑scale characterization within a compact structure. Three mineral samples Copper, Chrome, and Silica were analyzed using each fabricated sensor, demonstrating the sensor’s capability to differentiate materials based on their electromagnetic dielectric contrast. The second version of the sensor for chrome sample analysis is offered high Q-factor (1214) and best Figure of Merit (10). The combined advantages of compactness, low cost, and dual‑scale operation make the proposed sensor a strong candidate for non‑destructive material characterization. Additionally, the methodology presented in this study provides a foundation for developing portable, field‑deployable sensing devices tailored for rapid, on‑site evaluation of mining and mineral‑based materials.

Reviewer-3

===Comments& Response=============================================================

Comment#1 The manuscript presents the design, fabrication, and experimental validation of a dual-version microwave sensor based on a complementary split-ring resonator (CSRR) array for mining-based material characterization. The sensing structure employs a circular patch loaded with a 3×3 CSRR array combined with rectangular slots to generate resonance in the transmission coefficient. Two versions of the sensor are introduced, operating at approximately 1.5 GHz and 5.1 GHz respectively, with experimental validation performed using copper, chrome, and silica mineral samples. The study claims improved sensing capability through dual-scale resonance and structural compactness.

However, despite the practical implementation and experimental effort, the manuscript contains several fundamental limitations related to novelty, methodological rigor, reproducibility, and scholarly originality, as detailed below.

The proposed sensing principle is based on conventional CSRR/SRR microwave resonator sensing mechanisms, which are widely established for dielectric and material characterization. Previous studies have already demonstrated CSRR-based sensors for solid, liquid, and environmental material detection, including multi-resonance and compact sensing structures. CSRR-based microwave sensing has long been recognized as a standard approach for dielectric characterization using resonance shift mechanisms, which rely on electromagnetic field perturbation caused by material properties (Boybay, M. S., & Ramahi, O. M. (2012). Material characterization using complementary split-ring resonators. IEEE Transactions on instrumentation and Measurement, 61(11), 3039-3046.) The main contribution of the manuscript appears to be geometric scaling and dimensional optimization of known CSRR configurations rather than introducing a new sensing concept, analytical formulation, or algorithmic advancement. The claimed dual-version and dual-scale operation primarily reflects parameter resizing rather than methodological innovation.

Response#1 Thank you for your thorough and constructive feedback. We agree that CSRR‑based sensing principles are well established, and we appreciate the need to more clearly articulate the novelty of our contribution. While our work builds on conventional CSRR mechanisms, its primary innovation lies in the dual‑version, dual‑scale resonant architecture specifically optimized for mining‑grade solid material characterization, which has not been extensively explored in prior studies. The integration of a 3×3 CSRR array with targeted rectangular slot perturbations enables simultaneous low‑frequency at 1.5 GHz and high‑frequency 5.1 GHz sensitivity, allowing complementary penetration depth and surface‑level resolution beneficial for heterogeneous mineral samples. Additionally, the manuscript introduces a compact, fabrication‑friendly circular patch configuration, experimentally validated on three industrially relevant minerals, demonstrating practical tunability and enhanced sensing range. We have revised the introduction section last paragraph of the manuscript to more explicitly highlight these contributions, strengthen methodological clarity, and better position our study within the existing CSRR literature.

Comment#2 A major scholarly concern arises from the existence of earlier publications by the same research group that describe highly similar sensing concepts, structures, and applications.

For example, previously published work already reported mineral identification using a composite SRR-based microwave sensor designed for detecting minerals such as copper and chromite in similar geological contexts:

Sharma, M. K., Idries, N. K. S., Alkalbani, A. S., Satyanarayana, D., Rathinam, G., & Sharma, A. (2025). Minerals identification and characterization using a new 3D printed composite-structured split ring resonator microwave sensor. Discover Applied Sciences, 7(7), 757.

Additionally, related book chapter publications describing multi-resonating CSRR-based mining material characterization sensors appear to present substantially similar technical content and research objectives. The sensor structure, sensing mechanism, and mineral characterization experiments reported in those works strongly resemble the current submission:

Sharma, M. K., Idries, N. K. S., Alkalbani, A. S., Degala, S., Rathinam, G., & Sharma, A. (2025, July). A New Composite-Structured CSRR Loaded Multi-resonating Sensor for Mining Based Material Characterization. In International Conference on ICT for Sustainable Development (pp. 84-92). Cham: Springer Nature Switzerland.

Response#2 Thank you for this observation and for carefully reviewing the prior work from our research group. We acknowledge that the cited publications share thematic continuity with the current submission, as they all fall within our broader research program focused on developing resonator‑based microwave sensors for geological and mining applications. However, we would like to clarify several key distinctions that demonstrate the novelty and substantive advancement of the present study relative to the previously published work.

In the second cited paper: only simulation study of the proposed sensor was discussed by same research group, this study is extension of this conference paper and implemented sensor into physical form and conducted real experiments for mineral characterization.

Comment#3 The manuscript states that few studies have investigated mining-based material characterization. However, the literature review remains selective and does not provide systematic benchmarking or quantitative comparison showing performance improvement over existing CSRR sensors. The presented comparison table focuses mainly on geometry and operating frequency but omits key sensing performance metrics such as detection limit, measurement uncertainty, and repeatability.

Response#3 We appreciate the reviewer’s comment. In our revised manuscript, we clarify that, despite an extensive search, there is currently no directly comparable work in the specific area of mining‑based material characterization using CSRR sensors. Existing CSRR studies focus largely on industrial, chemical, or biological materials, and do not address mining applications or similar sensing conditions. For this reason, meaningful quantitative benchmarking using detection limit, repeatability, or uncertainty is not possible across prior studies. Instead, we emphasize the novelty of our work and provide detailed performance evaluation based on our experimental results, establishing a foundation for future comparative research in this emerging domain.

Kindly refer to third paragraph of introduction in revised manuscript for this justification.Same as given below:

Despite a comprehensive search of existing literature, no prior work has been reported in the specific domain of mining‑based material characterization employing CSRR sensors. Indeed, all the existing works on CSRR sensors have been carried out on industrial, chemical, and biological materials. Therefore, there is a limitation to execute quantitative comparison with the existing literature. However, we compared and discussed related literature of the resonating sensor with proposed work in Table-1. Instead, the novelty of the work is emphasized, and the performance evaluation is carried out in detail using the experimental results obtained.

Comment#4 The sensor design relies heavily on numerical optimization using CST simulation without providing a generalized theoretical or analytical design methodology.

Response#4 We thank the reviewer for this observation. Numerical optimization in CST is indeed central to CSRR‑based sensor development due to the complexity of electromagnetic behavior, and simulation‑driven refinement is often necessary to achieve high sensitivity and application‑specific performance. We acknowledge the importance of generalized analytical design methodologies, and this feedback is valuable for guiding future work toward broader theoretical frameworks.

Comment#5 The equivalent circuit model is presented but lacks parameter extraction validation or quantitative error comparison between circuit model and electromagnetic simulation.

Response#5 We thank the reviewer for this valuable observation. The equivalent circuit model in our work is primarily intended to offer a conceptual understanding of the sensor’s resonance behavior rather than to serve as a fully validated predictive model. We acknowledge that quantitative parameter extraction and an explicit error comparison with electromagnetic simulations would strengthen the analytical rigor.

Comment#6 Only three mineral materials are tested, which is insufficient to demonstrate general sensing applicability.

Response#6 We thank the reviewer for this comment. The selection of copper, chrome, and silica was intentional, as these materials represent key mineral resources of relevance to the Omani mining sector and therefore align directly with the intended application domain of the proposed sensor. Our objective was to evaluate sensor behavior using representative materials rather than to demonstrate universal applicability across all mineral types. We agree that testing a broader range of materials could further establish generalizability, and we consider this an important direction for future work building on the current application‑focused study.

Comment#7 The second optimized sensor version is validated using only one concentration level, weakening the claimed dual-scale sensing reliability.

Response#7 Thank you for highlighting this important point. We agree that validating the second optimized sensor version using a single concentration level could limit the perceived robustness of the dual‑scale sensing performance. In response, we have expanded the validation experiments by incorporating multiple concentration levels. These additional measurements are now included in the revised manuscript (see Section 4 and Figure 11), demonstrating consistent sensor behavior across the full targeted detection range. This extended dataset strengthens the reliability of the dual-scale sensing claim and provides a more comprehensive evaluation of the optimized sensor’s performance. Revised Fig.11 is shown below:

Fig.11. Experimental results of transmission coefficient (S21) for mining-based materials using optimized second version of the sensor

Comment#8 The results section mainly describes resonance shifts without providing deeper electromagnetic or physical interpretation.

Response#8 We thank the reviewer for the comment. The electromagnetic behavior and resonating principle of the sensor, including the electric‑field distribution analysis and the resonance‑shift formulation in Equation (1), are already explained in Section 2.1. However, to strengthen the discussion paragraph of optimized sensor (Section 4), we have now added sample analysis based on the quality factor, figure of merit, sensitivity. For this revision we included new tables (Table-2, Table-3, Table-4) for each mineral and same are given below:

The resonate frequency, minimum value of (S21), quality factor (Q), sensitivity, and Figure of Merit (FOM) for chrome, copper and silica samples are summarized in Table-2, Table-3, and Table-4 respectively. The results show that all chrome samples maintain a stable resonant response with a narrow 0.004 GHz bandwidth, producing highest (Q) factor for sample-3 i.e. (1214). While Sample 1 serves as the reference, Sample 2 and Sample 3 exhibit small but clear resonant frequency shifts, reflected in their sensitivities of 0.020 GHz and 0.040 GHz. These shifts demonstrate the sensor’s ability to detect incremental changes in the samples. Additionally, the (FOM) increases from 5.00 to 10.00 from Sample 2 to Sample 3, indicating improved sensing performance as the sensitivity rises while the resonance sharpness remains high.

Table-2 Key performance parameters for chrome samples

Sample Resonant Frequency (GHz) Min S21 (dB) Bandwidth (GHz) Quality Factor (Q) Sensitivity (GHz) FOM

Chrome@ Sample-1 4.836 -61.37 0.0040 1209.09 — —

Chrome@ Sample-2 4.816 -54.31 0.0040 1204.09 0.0200 5.00

Chrome@ Sample-3 4.856 -52.97 0.0040 1214.09 0.0400 10.00

The copper samples demonstrate a clear enhancement in sensing performance with increasing concentration, as evidenced by the resonance shift, decreasing bandwidth, and deeper (S21) values. The reduction in bandwidth leads to a higher (Q) factor, indicating sharper and more selective resonance. Simultaneously, sensitivity doubles and the (FOM) increased significantly, confirming improved detection capability.

Table-3 Key performance parameters for copper samples

Sample Resonant Frequency (GHz) Min S21 (dB) Bandwidth (GHz) Quality Factor (Q) Sensitivity (GHz) FOM

Copper@ Sample-1 4.752 -45.83 0.0100 475.19 — —

Copper@ Sample-2 4.722 -46.50 0.0080 590.26 0.0300 3.75

Copper@ Sample-3 4.782 -50.50 0.0060 796.99 0.0600 10.00

The silica samples show non-monotonic resonance behavior with concentration, indicating unstable sensor response. Although, bandwidth reduced at Sample-2, yielding a high Q (~1220), it increases significantly at Sample-3, causing Q degradation. The (S21) depth also varies inconsistently, reflecting non-uniform field interaction. While sensitivity increases, the (FOM) decreases at higher concentration due to bandwidth broadening, suggesting reduced sensing efficiency. In conclusion, silica demonstrated optimal performance only at intermediate concentration, with degraded reliability at higher loading.

Table-4 Key performance parameters for silica samples

Sample Resonant Frequency (GHz) Min S21 (dB) Bandwidth (GHz) Quality Factor (Q) Sensitivity (GHz) FOM

Silica@ Sample-1 4.896 -50.18 0.0060 816.06 — —

Silica@ Sample-2 4.880 -54.66 0.0040 1219.94 0.0160 4.00

Silica@ Sample-3 4.908 -46.39 0.0100 490.79 0.0280 2.80

A summary of the overall performance comparison for all three minerals tested using the optimized sensor is presented in Table‑5. The chrome measurements demonstrated the best overall performance, characterized by the highest Q-factor and (FOM), indicating extremely sharp resonance and superior sensing capability. In comparison, copper sample exhibited a strong and consistent progressive behavior, with sensing parameters such as Q-factor, sensitivity, and FOM improving steadily with increasing concentration, reflecting reliable and predictable performance. On the other hand, silica shows comparatively less reliability due to its inconsistent resonance shifts and fluctuating bandwidth, which lead to non-uniform Q-factor and reduced sensing efficiency.

Table-5 Overall performance comparison among three minerals results

Mineral Q-factor Best Sensitivity Best FOM Sensetivity

Copper High High High Good

Chrome Very High High Best Excellent

Silica Moderate Low Low Poor

=====================================================================

End of responses

Regards

Madan Kumar Sharma

Corresponding author

Madan.k@uob.edu.om

madansharma12@gmail.com

Attachment Submitted filename: Reply to Comments.docx

# Peer review: Decision Letter 1

**DOI:** 10.1371/journal.pone.0360251.r003

**Authors:** Prashanth S. Adarakatti

24 Aug 2026

Dear Dr. SHARMA,

Thank you for submitting your manuscript to PLOS One. After careful consideration, we feel that it has merit but does not fully meet PLOS One’s publication criteria as it currently stands. Therefore, we invite you to submit a revised version of the manuscript that addresses the points raised during the review process.

Please submit your revised manuscript by Oct 08 2026 11:59PM. If you will need more time than this to complete your revisions, please reply to this message or contact the journal office at plosone@plos.org. When you're ready to submit your revision, log on to https://www.editorialmanager.com/pone/ and select the 'Submissions Needing Revision' folder to locate your manuscript file.

- A letter that responds to each point raised by the academic editor and reviewer(s). You should upload this letter as a separate file labeled 'Response to Reviewers'.
- A marked-up copy of your manuscript that highlights changes made to the original version. You should upload this as a separate file labeled 'Revised Manuscript with Track Changes'.
- An unmarked version of your revised paper without tracked changes. You should upload this as a separate file labeled 'Manuscript'.

If you would like to make changes to your financial disclosure, please include your updated statement in your cover letter. Guidelines for resubmitting your figure files are available below the reviewer comments at the end of this letter.

If applicable, we recommend that you deposit your laboratory protocols in protocols.io to enhance the reproducibility of your results. Protocols.io assigns your protocol its own identifier (DOI) so that it can be cited independently in the future. For instructions see: https://journals.plos.org/plosone/s/submission-guidelines#loc-laboratory-protocols. Additionally, PLOS One offers an option for publishing peer-reviewed Lab Protocol articles, which describe protocols hosted on protocols.io. Read more information on sharing protocols at https://plos.org/protocols?utm_medium=editorial-email&utm_source=authorletters&utm_campaign=protocols.

As the corresponding author, your ORCID iD is verified in the submission system and will appear in the published article. PLOS supports the use of ORCID, and we encourage all coauthors to register for an ORCID iD and use it as well. Please encourage your coauthors to verify their ORCID iD within the submission system before final acceptance, as unverified ORCID iDs will not appear in the published article. *Only* the individual author can complete the verification step; PLOS staff *cannot* verify ORCID iDs on behalf of authors.

We look forward to receiving your revised manuscript.

Kind regards,

Prashanth S. Adarakatti

Academic Editor

PLOS One

Journal Requirements:

If the reviewer comments include a recommendation to cite specific previously published works, please review and evaluate these publications to determine whether they are relevant and should be cited. There is no requirement to cite these works unless the editor has indicated otherwise.

Please review your reference list to ensure that it is complete and correct. If you have cited papers that have been retracted, please include the rationale for doing so in the manuscript text, or remove these references and replace them with relevant current references. Any changes to the reference list should be mentioned in the rebuttal letter that accompanies your revised manuscript. If you need to cite a retracted article, indicate the article’s retracted status in the References list and also include a citation and full reference for the retraction notice.

Additional Editor Comments:

The present work describes the “Dual-Version and Dual-Scale CSRR Loaded Sensor for

Mining-Based Material Characterization”. The proposed work can be accepted only after answering few queries.

Here are my remarks:

1) How were variations in sample particle size, packing density, moisture content, and sample placement controlled to ensure reproducible sensing responses?

2) What is the specific advantage of using both scales compared with a single optimized sensor for mineral characterization?

3) Please clarify the equations used and explain whether these parameters are directly comparable across different resonant frequencies and sensor dimensions.

4) Have the authors evaluated its performance using real heterogeneous mineral samples containing impurities rather than only selected laboratory-prepared materials?

5) To enhance the manuscript's impact and appeal to a broader scientific readership, consider incorporating recent references to ensure completeness. These references should be included in the Introduction or other relevant sections as appropriate. Additionally, avoid citing more than five references within a single sentence to maintain clarity and readability.

Authors are encouraged to write the sentences below in the introduction section, incorporating the citations to enhance broader appeal and readability.

In order to provide dependable material characterization and monitoring, recent developments in sensor technology have shown the significance of multi-sensor dependency modeling, flexible sensing platforms, wireless sensor designs, and enhanced calibration techniques [1-3]. Multi-scale sensing approaches are becoming increasingly important, as evidenced by the development of high-precision cross-scale characterisation techniques for complicated three-dimensional objects [4]. Additionally, sensor array accuracy and reliability have been enhanced by directionally regularized calibration techniques and physics-inspired error modeling [5]. The adaptability of modern sensing systems for high-frequency and condition-monitoring applications is further demonstrated by recent advancements in time-frequency integration architectures and planar magnetically linked current sensors [6,7]. In mining-related settings, image-processing-based techniques have also been effectively used for material identification and classification [8]. Furthermore, guided-surface lattice resonances and high-Q resonant structures have garnered significant interest because of their acute resonance features and increased sensitivity [9]. For cutting-edge industrial applications, sustainable material-based sensing and prediction techniques are also developing [10]. The creation of the suggested dual-version and dual-scale CSRR-loaded sensor for mining-based material characterisation is supported by the collective findings of these research, which demonstrate the increasing demand for small, precise, high-Q, and multi-scale sensing platforms.

1) doi: https://doi.org/10.1016/j.aei.2025.104132

2) doi: https://doi.org/10.1016/j.mtadv.2025.100641

3) DOI:10.59277/romjist.2025.4.03

4) https://doi.org/10.1002/smtd.202501775

5) doi: 10.1109/TIM.2026.3660392

6) doi: 10.1109/TPEL.2025.3597418

7) doi: 10.1088/1361-6501/ae4130

8) https://doi.org/10.1080/19392699.2025.2481147

9) doi: 10.1038/s41467-026-71583-w

10) doi: https://doi.org/10.1016/j.triboint.2025.111324

[Note: HTML markup is below. Please do not edit.]

Reviewers' comments:

Reviewer's Responses to Questions

**Comments to the Author**

Reviewer #2: All comments have been addressed

Reviewer #3: (No Response)

**********

2. Is the manuscript technically sound, and do the data support the conclusions?

Reviewer #2: Yes

Reviewer #3: No

**********

3. Has the statistical analysis been performed appropriately and rigorously?

Reviewer #2: N/A

Reviewer #3: No

**********

4. Have the authors made all data underlying the findings in their manuscript fully available?

The PLOS Data policy

Reviewer #2: Yes

Reviewer #3: Yes

**********

5. Is the manuscript presented in an intelligible fashion and written in standard English?

Reviewer #2: Yes

Reviewer #3: Yes

**********

Reviewer #2: (No Response)

Reviewer #3: When comparing the revised manuscript with the previous version, the authors have improved the presentation by revising the introduction, figures, conclusion, and adding some performance metrics. However, the major concerns remain insufficiently addressed.

The most critical unresolved issue is the relationship between the present manuscript and the authors’ previously published conference/book chapter paper: Sharma et al., “A New Composite-Structured CSRR Loaded Multi-resonating Sensor for Mining Based Material Characterization,” ICT4SD 2025, Springer. The authors acknowledge that the earlier paper presented the same proposed sensor concept and that the current manuscript is an extension involving fabrication and experimental validation. However, no detailed comparison is provided to clearly distinguish previously published material from genuinely new scientific content. Therefore, concerns regarding substantial overlap, incremental publication, and insufficient originality remain unresolved.

In addition, the novelty remains limited, as the work is mainly based on well-established CSRR/SRR microwave sensing principles. The experimental validation is also still limited, with insufficient repeatability, uncertainty analysis, statistical evaluation, and quantitative comparison with existing methods.

**********

what does this mean?). If published, this will include your full peer review and any attached files.

If you choose “no”, your identity will remain anonymous but your review may still be made public.

**Do you want your identity to be public for this peer review?** For information about this choice, including consent withdrawal, please see our Privacy Policy

Reviewer #2: **Yes:**  Santanu Kumar Behera

Reviewer #3: No

**********

[NOTE: If reviewer comments were submitted as an attachment file, they will be attached to this email and accessible via the submission site. Please log into your account, locate the manuscript record, and check for the action link "View Attachments". If this link does not appear, there are no attachment files.]

To ensure your figures meet our technical requirements, please review our figure guidelines: https://journals.plos.org/plosone/s/figures

You may also use PLOS’s free figure tool, NAAS, to help you prepare publication quality figures: https://journals.plos.org/plosone/s/figures#loc-tools-for-figure-preparation.

NAAS will assess whether your figures meet our technical requirements by comparing each figure against our figure specifications.

# Author comment: Author response to Decision Letter 2

**DOI:** 10.1371/journal.pone.0360251.r004

2 Sep 2026

Submission ID: PONE-D-25-62986

Original Article Title: " Dual-Version and Dual-Scale CSRR Loaded Sensor for Mining-Based Material Characterization"

To: Submission ID PONE-D-25-62986 (Revision-2)

Re: Response to reviewers

Dear Editor-in-Chief,

Thank you for allowing the resubmission of our manuscript, with an opportunity to address the reviewers’ comments.

Best regards,

Madan Kumar Sharma

Author response:

We would like to thank the Chief Editor, and Editor of PLOS One journal for having provided the helpful review report, and for having given us a chance for doing revision of the manuscript. Based on comments and suggestions from the reviewers, we have incorporated points as suggested by respected reviewer and editor. We hope the editor and the reviewers will be satisfied with these revisions. Kindly refer to the following sections for these responses.

We are uploading two files, (a) our point-by-point response to the comments (below) (response to reviewers), (b) an updated manuscript with indicating changes in response to all reviewer’s comments with highlighted different colors, (c) Clean copy of the manuscript.

Few important points regarding revisions are addressed below:

1. Comments & responses to Editor are addressed by green color font.

2. Comments & responses to reviewer #3 are addressed by blue color font.

3. Updated tables and table numbers as below:

Previous Revised Remark

Table-1 - As it is

Table-2 Added new table on recommendation of reviewer comment.

Table-2 to Table-5 Table-3 to Table-6 Only order is updated.

======================================================================

Editor Comments & Response

===Comments& Response=============================================================

======================================================================

In addition to reviewer comment:

Comment#1

How were variations in sample particle size, packing density, moisture content, and sample placement controlled to ensure reproducible sensing responses?

Response#1

Thanks for raising this very important point. In order to obtain reproducible responses from the sensors, all the samples were prepared and tested under the same conditions. The particle size distribution was kept constant by sieving the sample through the same mesh size before carrying out the measurements. The packing density was ensured by keeping the mass of the sample as well as the loading method same for all the tests. Variations due to moisture content were taken care of by drying the samples under controlled conditions before carrying out the measurements and then storing them in a desiccator. Moreover, the samples were placed in the same place inside the sensing chamber.

Comment#2

What is the specific advantage of using both scales compared with a single optimized sensor for mineral characterization?

Response#2

Thank you for this insightful comment. The key advantage of the dual-frequency sensor is that it captures dielectric responses at two different frequency scales, providing complementary information that cannot be obtained from a single-frequency sensor. Since mining materials exhibit frequency-dependent dielectric characteristics, the lower-frequency resonance probes bulk properties while the higher-frequency resonance offers greater sensitivity to subtle compositional variations. Consequently, the dual-frequency approach improves material discrimination, dielectric-property estimation accuracy, and overall characterization reliability compared to a single optimized sensor.

Comment#3

Please clarify the equations used and explain whether these parameters are directly comparable across different resonant frequencies and sensor dimensions.

Response#3

Thank you for this valuable comment. These equations describe the fundamental sensing parameters derived from the resonant response of the microwave sensor and are not restricted to a particular resonance mode or operating frequency. Therefore, Equations (1) and (2) are applicable to each resonant frequency exhibited by the sensor, including both frequency bands considered in this work.

Regarding parameter comparison across different resonant frequencies and sensor dimensions, the extracted quantities can be directly compared only when they are appropriately normalized or interpreted within the context of their corresponding resonance mode. Since resonant frequency, quality factor, and field distribution are inherently dependent on the sensor geometry and operating frequency, the absolute values of the measured responses may vary between different sensor dimensions or resonance bands. Nevertheless, Equations (1) and (2) remain valid for all resonances because they are based on the same underlying perturbation mechanism relating the resonant characteristics to the dielectric properties of the material under test.

Comment#4

Have the authors evaluated its performance using real heterogeneous mineral samples containing impurities rather than only selected laboratory-prepared materials?

Response#4

Thank you for this important comment. Yes, the proposed sensor was evaluated using real mining-derived mineral samples containing naturally occurring impurities and compositional variations, rather than being limited to idealized laboratory-prepared materials. These samples inherently exhibit heterogeneity in terms of mineral composition, impurity content, particle morphology, and dielectric characteristics, thereby providing a more realistic assessment of sensor performance.

The experimental results demonstrated that the sensor was capable of producing distinct and repeatable responses for the tested samples despite their inherent variability. This indicates that the proposed dual-frequency sensing approach is sufficiently robust to characterize practical mining materials and is not limited to controlled laboratory conditions. We have clarified this aspect in the revised manuscript and provided additional details regarding the origin and characteristics of the tested mineral samples.

Comment#5

To enhance the manuscript's impact and appeal to a broader scientific readership, consider incorporating recent references to ensure completeness. These references should be included in the Introduction or other relevant sections as appropriate. Additionally, avoid citing more than five references within a single sentence to maintain clarity and readability.

Response#5

We sincerely thank the reviewer for this valuable suggestion. In response, we have carefully reviewed the recent literature and incorporated several up-to-date and relevant references throughout the manuscript, particularly in the Introduction and discussion sections, to improve the completeness and broader scientific context of our study. Furthermore, we have revised the citation style to avoid citing more than five references within a single sentence. Where necessary, references have been redistributed across multiple sentences to enhance clarity, readability, and overall presentation of the manuscript. We believe these revisions have strengthened the manuscript and improved its accessibility to a wider scientific audience. Newly latest references are given below:

[6] M. K. Sharma, A. S. Alkalbani, S. B. M. Sulaiman, and S. Degala, “Low-cost disposable HSRR-biosensor for skin disease characterization and deep-learning assist decisions,” Journal of Hazardous Materials Advances, vol. 23, p. 101327, Aug. 2026, doi: 10.1016/J.HAZADV.2026.101327.

[7] W. Liu and Z. Su, “Multi-band microwave sensor for agricultural microsample analysis using dual asymmetric metamaterial resonators toward enhanced and balanced full-band sensitivity,” Comput. Electron. Agric., vol. 250, p. 111839, Aug. 2026, doi: 10.1016/J.COMPAG.2026.111839.

[8] M. A. Aldhaeebi, A. Ali, and T. S. Almoneef, “A Machine Learning-Augmented Microwave Sensor for Metallic Landmine Detection,” Signals 2026, Vol. 7, Page 40, vol. 7, no. 3, p. 40, May 2026, doi: 10.3390/SIGNALS7030040.

Comment#6 Authors are encouraged to write the sentences below in the introduction section, incorporating the citations to enhance broader appeal and readability.

Response#6 Thank you for your kind suggestion. As written text in comment letter has been incorporated revised manuscript and same given below including recommended citations.

In order to provide dependable material characterization and monitoring, recent developments in sensor technology have shown the significance of multi-sensor dependency modeling, flexible sensing platforms, wireless sensor designs, and enhanced calibration techniques [20], [21], [22]. Multi-scale sensing approaches are becoming increasingly important, as evidenced by the development of high-precision cross-scale characterization techniques for complicated three-dimensional objects [23]. Additionally, sensor array accuracy and reliability have been enhanced by directionally regularized calibration techniques and physics-inspired error modeling [24] . The adaptability of modern sensing systems for high-frequency and condition-monitoring applications is further demonstrated by recent advancements in time-frequency integration architectures and planar magnetically linked current sensors[25], [26] . In mining-related settings, image-processing-based techniques have also been effectively used for material identification and classification [27]. Furthermore, guided-surface lattice resonances and high-Q resonant structures have garnered significant interest because of their acute resonance features and increased sensitivity [28]. For cutting-edge industrial applications, sustainable material-based sensing and prediction techniques are also developed in [29]. The creation of the suggested dual-version and dual-scale CSRR-loaded sensor for mining-based material characterization is supported by the collective findings of this research, which demonstrate the increasing demand for small, precise, high-Q, and multi-scale sensing platforms.

Reviwer#2

===Comments& Response=================================================================

Comment: All comments have been addressed.

Author’s Response Thank you for your valuable time and reviewing this manuscript and provide your constructive feedback on it.

Reviewer-3

===Comments& Response===================================================================

When comparing the revised manuscript with the previous version, the authors have improved the presentation by revising the introduction, figures, conclusion, and adding some performance metrics. However, the major concerns remain insufficiently addressed.

Author’s Response:

We sincerely thank the reviewer for acknowledging the improvements made in the revised manuscript. We also appreciate the observation that some of the major concerns were not sufficiently addressed in the previous revision. In response, we have carefully re-examined all comments and made substantial additional revisions to strengthen the manuscript.

Comment#1

The most critical unresolved issue is the relationship between the present manuscript and the authors’ previously published conference/book chapter paper: Sharma et al., “A New Composite-Structured CSRR Loaded Multi-resonating Sensor for Mining Based Material Characterization,” ICT4SD 2025, Springer. The authors acknowledge that the earlier paper presented the same proposed sensor concept and that the current manuscript is an extension involving fabrication and experimental validation. However, no detailed comparison is provided to clearly distinguish previously published material from genuinely new scientific content. Therefore, concerns regarding substantial overlap, incremental publication, and insufficient originality remain unresolved.

Response#1

We sincerely thank the reviewer for highlighting this important concern regarding the relationship between the current manuscript and our previously published conference/book chapter paper (Sharma et al., ICT4SD 2025, Springer). We fully agree that a clear distinction between the previously published work and the novel contribution of the present manuscript is essential to avoid any ambiguity regarding originality and scientific contribution.

In contrast, the present manuscript substantially extends that work through several new contributions, including:

1.Fabrication and Experimental Validation: The sensor has been physically fabricated and experimentally characterized, which was not included in the conference/book chapter publication.

2.Comprehensive Measurement-Based Analysis: Detailed experimental investigations have been carried out to validate the sensing performance under practical conditions, including comparison between simulated and measured results.

3.Extended Material Characterization Study: The present work demonstrates the applicability of the proposed sensor for characterization of mining-related materials using real measurements, providing insights that were not available in the previous publication.

4.Additional Performance Evaluation: New performance metrics, sensitivity analysis, and validation results have been incorporated to establish the robustness and practical effectiveness of the proposed sensor.

5.Significantly Expanded Discussion and Interpretation: The manuscript includes a more comprehensive analysis of the sensing mechanism, experimental observations, limitations, and potential real-world applications.

To ensure transparency, we have added a clear comparison table (Table 2) in the revised manuscript summarizing the scope, methodology, and contributions of both publications. We have also revised the introduction and related sections to explicitly acknowledge the prior work and clearly state the novel scientific contributions presented in the current study.

Comment#2 In addition, the novelty remains limited, as the work is mainly based on well-established CSRR/SRR microwave sensing principles. The experimental validation is also still limited, with insufficient repeatability, uncertainty analysis, statistical evaluation, and quantitative comparison with existing methods.

Response#3

We sincerely thank the reviewer for this insightful comment. We acknowledge that CSRR/SRR-based microwave sensing is a well-established research area. However, the novelty of the present work lies not merely in the use of the CSRR/SRR sensing principle, but in the proposed sensor configuration, its optimization strategy, and its application methodology, which together provide improved sensing performance compared with conventional designs. However, quantitative analysis is done in terms of quality factor (Q-factor), sensitivity, FOM. We included Q-factor in the revised comparison table-1, compared with existing methods. Other quantitative parameters are not completely available in the literature so that unable to include in this comparative study.

We believe that these revisions substantially strengthen the manuscript by providing a clearer demonstration of its novelty, improving the rigor of the experimental validation, and enhancing the overall scientific contribution of the work.

=====================================================================

End of responses

Regards

Madan Kumar Sharma

Corresponding author

Madan.k@uob.edu.om

madansharma12@gmail.com

Attachment Submitted filename: Response to Reviewers.docx

# Editor report: Decision Letter 2

**DOI:** 10.1371/journal.pone.0360251.r005

**Authors:** Prashanth S. Adarakatti

25 Sep 2026

Dual-Version and Dual-Scale CSRR Loaded Sensor for Mining-Based Material Characterization

PONE-D-25-62986R2

Dear Madan Kumar Sharma,

We’re pleased to inform you that your manuscript has been judged scientifically suitable for publication and will be formally accepted for publication once it meets all outstanding technical requirements.

Within one week, you’ll receive an e-mail detailing the required amendments. When these have been addressed, you’ll receive a formal acceptance letter and your manuscript will be scheduled for publication.

An invoice will be generated when your article is formally accepted. Please note, if your institution has a publishing partnership with PLOS and your article meets the relevant criteria, all or part of your publication costs will be covered. Please make sure your user information is up-to-date by logging into Editorial Manager at Editorial Manager® and clicking the ‘Update My Information' link at the top of the page. For questions related to billing, please contact billing support.

If your institution or institutions have a press office, please notify them about your upcoming paper to help maximize its impact. If they’ll be preparing press materials, please inform our press team as soon as possible -- no later than 48 hours after receiving the formal acceptance. Your manuscript will remain under strict press embargo until 2 pm Eastern Time on the date of publication. For more information, please contact onepress@plos.org.

Kind regards,

Prashanth S. Adarakatti

Academic Editor

PLOS One

Additional Editor Comments (optional):

Accept.

Reviewers' comments:

# Editor report: Acceptance letter

**DOI:** 10.1371/journal.pone.0360251.r006

**Authors:** Prashanth S. Adarakatti

PONE-D-25-62986R2

PLOS One

Dear Dr. SHARMA,

I'm pleased to inform you that your manuscript has been deemed suitable for publication in PLOS One. Congratulations! Your manuscript is now being handed over to our production team.

At this stage, our production department will prepare your paper for publication. This includes ensuring the following:

* All references, tables, and figures are properly cited

* All relevant supporting information is included in the manuscript submission,

* There are no issues that prevent the paper from being properly typeset

You will receive further instructions from the production team, including instructions on how to review your proof when it is ready. Please keep in mind that we are working through a large volume of accepted articles, so please give us a few days to review your paper and let you know the next and final steps.

Lastly, if your institution or institutions have a press office, please let them know about your upcoming paper now to help maximize its impact. If they'll be preparing press materials, please inform our press team within the next 48 hours. Your manuscript will remain under strict press embargo until 2 pm Eastern Time on the date of publication. For more information, please contact onepress@plos.org.

You will receive an invoice from PLOS for your publication fee after your manuscript has reached the completed accept phase. If you receive an email requesting payment before acceptance or for any other service, this may be a phishing scheme. Learn how to identify phishing emails and protect your accounts at https://explore.plos.org/phishing.

If we can help with anything else, please email us at customercare@plos.org.

Thank you for submitting your work to PLOS One and supporting open access.

Kind regards,

PLOS One Editorial Office Staff

on behalf of

Dr. Prashanth S. Adarakatti

Academic Editor

PLOS One
